# A Comprehensive Functional Portrait of Two Heat Shock Factor-Type Transcriptional Regulators Involved in *Candida albicans* Morphogenesis and Virulence

**DOI:** 10.1371/journal.ppat.1003519

**Published:** 2013-08-15

**Authors:** Sadri Znaidi, Audrey Nesseir, Murielle Chauvel, Tristan Rossignol, Christophe d'Enfert

**Affiliations:** 1 Institut Pasteur, Unité Biologie et Pathogénicité Fongiques, Département Génomes et Génétique, Paris, France; 2 INRA, USC2019, Paris, France; 3 Université Paris Diderot, Sorbonne Paris Cité, Cellule Pasteur, Paris, France; Geisel School of Medicine at Dartmouth, United States of America

## Abstract

Sfl1p and Sfl2p are two homologous heat shock factor-type transcriptional regulators that antagonistically control morphogenesis in *Candida albicans*, while being required for full pathogenesis and virulence. To understand how Sfl1p and Sfl2p exert their function, we combined genome-wide location and expression analyses to reveal their transcriptional targets *in vivo* together with the associated changes of the *C. albicans* transcriptome. We show that Sfl1p and Sfl2p bind to the promoter of at least 113 common targets through divergent binding motifs and modulate directly the expression of key transcriptional regulators of *C. albicans* morphogenesis and/or virulence. Surprisingly, we found that Sfl2p additionally binds to the promoter of 75 specific targets, including a high proportion of hyphal-specific genes (HSGs; *HWP1*, *HYR1*, *ECE1*, others), revealing a direct link between Sfl2p and hyphal development. Data mining pointed to a regulatory network in which Sfl1p and Sfl2p act as both transcriptional activators and repressors. Sfl1p directly represses the expression of positive regulators of hyphal growth (*BRG1*, *UME6*, *TEC1*, *SFL2*), while upregulating both yeast form-associated genes (*RME1*, *RHD1*, *YWP1*) and repressors of morphogenesis (*SSN6*, *NRG1*). On the other hand, Sfl2p directly upregulates HSGs and activators of hyphal growth (*UME6*, *TEC1*), while downregulating yeast form-associated genes and repressors of morphogenesis (*NRG1*, *RFG1*, *SFL1*). Using genetic interaction analyses, we provide further evidences that Sfl1p and Sfl2p antagonistically control *C. albicans* morphogenesis through direct modulation of the expression of important regulators of hyphal growth. Bioinformatic analyses suggest that binding of Sfl1p and Sfl2p to their targets occurs with the co-binding of Efg1p and/or Ndt80p. We show, indeed, that Sfl1p and Sfl2p targets are bound by Efg1p and that both Sfl1p and Sfl2p associate *in vivo* with Efg1p. Taken together, our data suggest that Sfl1p and Sfl2p act as central “switch on/off” proteins to coordinate the regulation of *C. albicans* morphogenesis.

## Author Summary

*Candida albicans* can switch from a harmless colonizer of body organs to a life-threatening invasive pathogen. This switch is linked to the ability of *C. albicans* to undergo a yeast-to-filament shift induced by various cues, including temperature. Sfl1p and Sfl2p are two transcription factors required for *C. albicans* virulence, but antagonistically regulate morphogenesis: Sfl1p represses it, whereas Sfl2p activates it in response to temperature. We show here that Sfl1p and Sfl2p bind *in vivo*, via divergent motifs, to the regulatory region of a common set of targets encoding key determinants of morphogenesis and virulence and exert both activating and repressing effects on gene expression. Additionally, Sfl2p binds to specific targets, including genes essential for hyphal development. Bioinformatic analyses suggest that Sfl1p and Sfl2p control *C. albicans* morphogenesis by cooperating with two important regulators of filamentous growth, Efg1p and Ndt80p, a premise that was confirmed by the observation of concomitant binding of Sfl1p, Sfl2p and Efg1p to the promoter of target genes and the demonstration of direct or indirect physical association of Sfl1p and Sfl2p with Efg1p, *in vivo*. Our data suggest that Sfl1p and Sfl2p act as central “switch on/off” proteins to coordinate the regulation of *C. albicans* morphogenesis.

## Introduction

*Candida albicans* is the most frequent causative agent of superficial as well as disseminated, life-threatening fungal infections [1]. The success of *C. albicans* as a major fungal pathogen of humans relies on a number of pathogenic traits, among which its capacity to grow and switch between at least three distinctive morphological forms: budding yeast, pseudohyphae and hyphae [2]–[5]. The morphogenetic transition has been commonly described as a critical trait for survival and virulence in the host, even though the analysis of a wide array of *C. albicans* knock-out mutants suggests that pathogenesis can be dissociated to some extent from morphological switching [6]–[8].

The yeast-to-hyphae transition is triggered by a variety of environmental stimuli including nutrient availability, temperature, pH, CO_2_ and serum [9]–[13]. This process correlates with the coordinated expression of a set of hyphal-specific genes (HSGs) with roles in orchestrating hyphal development. Consequently, the transition is highly regulated and involves multiple interconnected signalling pathways, including the cyclic AMP-dependent Protein Kinase A (cAMP-PKA, regarded as playing a central role in the control of morphogenesis), the Cph1p-mediated Mitogen-Activated Protein Kinase (MAPK) and the Rim101p-mediated pH cascade pathways, all of which positively regulate hyphal development through the modulation of the activity of transcription factors to control the expression of HSGs (see [13] for a recent review). These transcription factors include (among others) Efg1p/Flo8p, acting downstream of cAMP-PKA [14]–[20], Tec1p [21] and Ume6p [22], [23]. Hyphal morphogenesis is also subject to negative regulation mostly by the general corepressor Tup1p through interaction with the transcriptional repressors Nrg1p and Rfg1p [4], [12], [24]–[27].

In the yeast *Saccharomyces cerevisiae*, which has been used as a model for studying the transcriptional control of the morphological transition [28], [29], Sfl1p (ScSfl1p, for suppressor gene for flocculation 1) is a target of the cAMP-PKA pathway [30]. Sc*SFL1* encodes a negative regulator of pseudohyphal growth and invasion [31] and was isolated based on its ability to suppress flocculation defects in yeast [32]. ScSfl1p carries a putative heat shock factor (HSF)-type DNA binding domain and binds *in vitro* to a GAA triplet motif [33] characteristic of heat shock elements (HSEs) [34], while exerting its negative regulation through the recruitment of the Ssn6p-Tup1p corepressor complex [35]. ScSfl1p has dual activator/repressor functions, acting as a transcriptional repressor of flocculation-related genes and as an activator of stress-responsive genes [35], [36]. Interestingly, the *C. albicans* genome encodes two structural homologs of ScSfl1p, namely Sfl1p and Sfl2p [37]–[40]. Either *SFL1* or *SFL2* functionally complement an *S. cerevisiae sfl1* mutation [38], [39] and encode important regulators of morphogenesis and virulence in *C. albicans* [37]–[40]. Intriguingly, although sharing structural homologies, Sfl1p and Sfl2p have antagonistic functions: while Sfl1p acts as a negative regulator of hyphal development, Sfl2p acts as a positive regulator of this process [37]–[40]. Functional analyses of *C. albicans* Sfl1p showed that deletion of *SFL1* promoted filamentous growth and cell flocculation and correlated with induction of HSGs (*ECE1*, *HWP1*) and genes involved in cell adhesion (*ALS1*, *ALS3*), whereas its overexpression inhibited hyphal formation [37], [38]. Consistent with a transcriptional regulatory function, an Sfl1p-GFP fusion localized to the nucleus, while one hybrid *lacZ* reporter analyses in *C. albicans* correlated with a repressor function [37]. Importantly, either deletion or overexpression of *SFL1* attenuated *C. albicans* virulence in a mouse model of systemic infection [38]. On the other hand, we and others have shown that deletion of *SFL2* impaired filamentation in response to different cues, whereas *SFL2* overexpression promoted hyphal growth, even under non hyphae-stimulating conditions [39]–[41]. Noteworthy, an *sfl2*Δ/*sfl2*Δ strain exhibited reduced damage in a reconstituted human oral epithelium model and displayed attenuated virulence in a mouse model of gastrointestinal colonization and dissemination model [39], [40], indicating that Sfl2p also plays an important role in *C. albicans* pathogenesis. Similar to Sfl1p, an Sfl2p-GFP fusion localized to the nucleus, in line with a role in transcriptional regulation [39].

It is still unknown how Sfl1p and Sfl2p exert their antagonistic functions. Both *SFL1* and *SFL2* were shown to genetically interact with at least transcription factor *FLO8*. Hyphal development in *sfl1*Δ/*sfl1*Δ was abolished upon deletion of *FLO8* but enhanced upon *FLO8* overexpression [38] while overexpression of *SFL2* triggered filamentation in a *FLO8*- and *EFG1*-dependent manner [39], suggesting the implication of the cAMP-PKA pathway. It was also shown that *SFL2* is required for hyphal maintenance at high temperature and that a temperature increase from 25°C to 37°C leads to upregulation of both the RNA and protein levels of Sfl2p, indicating that Sfl2p is a temperature-responsive regulator [39]. In contrast, no clear association was determined between temperature and Sfl1p function. Interestingly, Song *et al.* showed that the putative HSF domains of Sfl1p and Sfl2p were required for their functional divergence by testing HSF domain-swapped hybrids for their ability to retain their effect on filamentation [39]. This suggests that the two putative HSF domains in Sfl1p and Sfl2p mediate the specific recognition of divergent target sites that determine the activation or repression roles of Sfl1p and Sfl2p [39]. To shed more light on Sfl1p and Sfl2p functions, we provide a comprehensive functional portrait of these two regulators using a combination of genome-wide location, genome-wide expression and genetic interaction analyses. We provide evidences that Sfl1p and Sfl2p act as central “switch on-off” proteins to coordinate the regulation of *C. albicans* morphogenesis and, potentially, pathogenesis and virulence.

## Results

### Epitope-tagging of Sfl1p and Sfl2p

**Figure 1:**
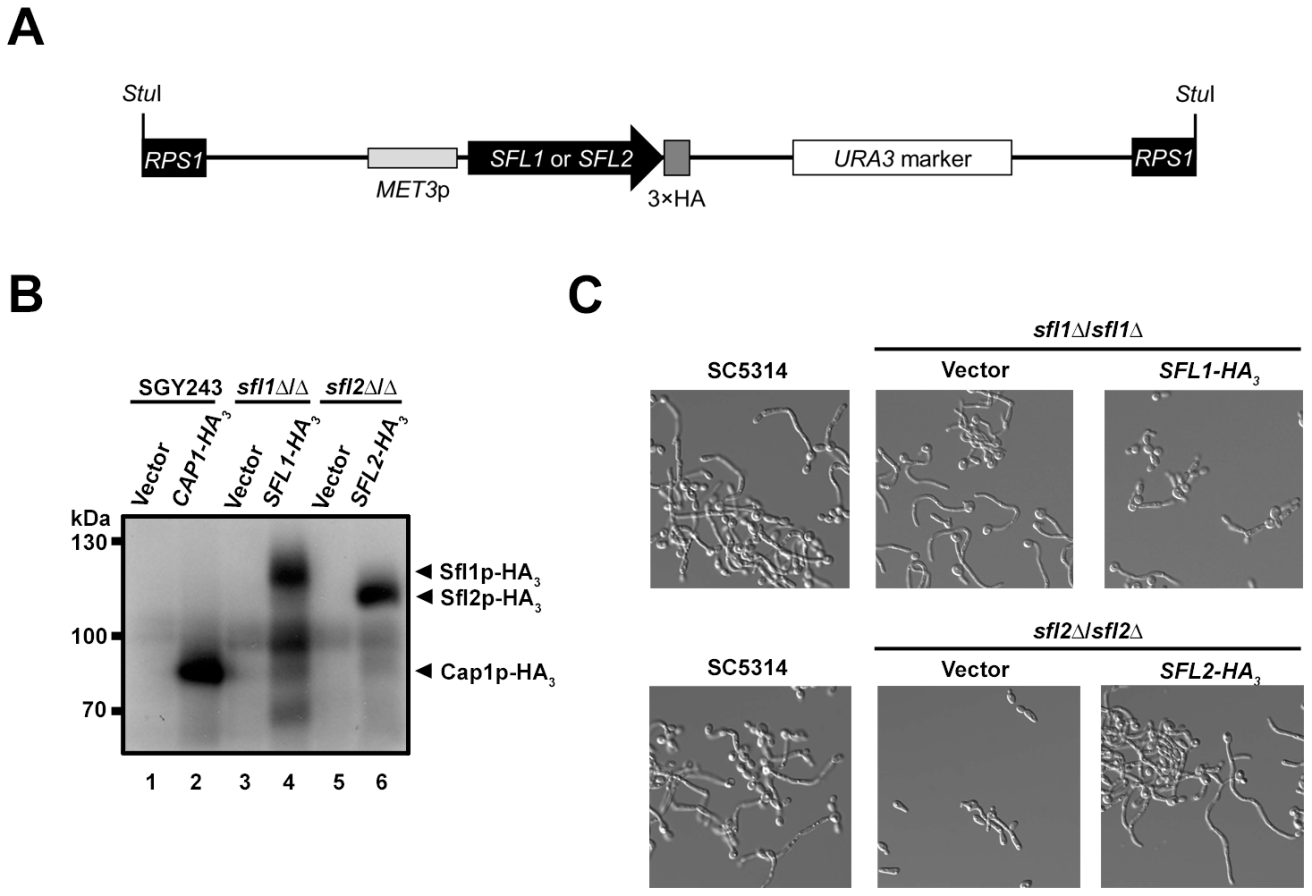
Strategy for tagging Sfl1p and Sfl2p with a triple hemagglutinin (3×HA) epitope tag and characterization of the tagged strains. (**A**) Schematic representation of the *SFL1-HA_3_* or *SFL2-HA_3_* tagging cassette allowing expression of the Sfl1p-HA_3_ or Sfl2p-HA_3_ fusion proteins following a *Stu*I digestion (*Stu*I) and integration at the *RPS1* locus (*RPS1*, black rectangles) [42]. A triple HA tag (dark grey box) was inserted in frame with the *SFL1* or *SFL2* coding sequences (*SFL1* or *SFL2*; black arrowed rectangle) in plasmid pCaEXP [42]. The tagged alleles are placed under the control of the *MET3* promoter (*MET3*p; ligh grey rectangle), which is induced in the absence of methionine and cysteine, and are followed by the *C. albicans URA3* marker (open rectangle). (**B**) Western blot analysis of homozygous *sfl1* or *sfl2* mutants (*sfl1*Δ/*sfl1*Δ or *sfl2*Δ/*sfl2*Δ) expressing HA_3_-tagged versions of the *SFL1* or *SFL2* genes, respectively (*SFL1-HA_3_* or *SFL2- HA_3_*) together with the corresponding empty vector controls (Vector). The SGY243 strain expressing the *CAP1-HA_3_* (*CAP1-HA_3_*) or carrying the empty vector (Vector) were used as a positive control [43]. Strains were grown overnight in SD medium (P*_MET3_*-inducing conditions) and total protein extracts were prepared then subjected to SDS-PAGE. Western blotting was performed using an anti-HA antibody. Positions of the molecular mass standards are indicated on the left (kDa). Immunopositive signals from the Sfl1p-HA_3_ and Sfl2p-HA_3_ fusions are indicated with black arrows (**C**) Phenotypic analysis of the strains expressing the HA_3_-tagged *SFL1* or *SFL2* alleles. Strain SC5314 (control) together with the homozygous *sfl1* or *sfl2* mutants expressing the *SFL1-HA_3_* or *SFL2-HA_3_* alleles (*SFL1-HA_3_*, *SFL2-HA_3_*), respectively, or carrying the empty vector (Vector) were grown overnight in YPD at 30°C then transferred to Lee's medium lacking methionine and cysteine and allowed to grow during 4 h at 37°C before being examined microscopically (40× magnification).

To better characterize the function of Sfl1p and Sfl2p, we sought to identify their DNA-binding locations, *in vivo*, by chromatin immunoprecipitation. To this end, we generated triple-hemagglutinin epitope (HA_3_)-tagged versions of *SFL1* and *SFL2* and used the pCaEXP system [42] to drive *MET3* promoter-dependent expression of the tagged alleles in *sfl1*Δ/*sfl1*Δ (Table 1; strain *sfl1*-CaEXP-*SFL1-HA_3_*) and *sfl2*Δ/*sfl2*Δ (Table 1, strain *sfl2*-CaEXP-*SFL2-HA_3_*) mutant strains, respectively (Figure 1A, see Materials and Methods for specific details). We also generated *sfl1*Δ/*sfl1*Δ and *sfl2*Δ/*sfl2*Δ mutants carrying the empty pCaEXP vector (*sfl1*-CaEXP and *sfl2*-CaEXP, respectively, see Table 1) to serve as negative controls for immunoprecipitation. Western blot analyses of strains grown under P*_MET3_*-inducing conditions showed that both Sfl1p-HA_3_ and Sfl2p-HA_3_ fusion proteins were expressed (Figure 1B, lanes 4 and 6). As an additional control for signal specificity, immunoblotting of total extracts from a *C. albicans* strain expressing the Cap1p-HA_3_ fusion (Figure 1, lane 2) or the corresponding empty-vector negative control (Figure 1, lane 1) was used [43].

**Table 1:** Strains used in this study.

Strain name	Lab identifier	Parental strain	Relevant genotype	Reference
SC5314	CEC1462		Prototrophic	[84]
CAI4	CEC2095	SC5314	*ura3*Δ::*λimm434*/*ura3*Δ::*λimm434*	[85]
BWP17H	CEC157	BWP17	*ura3*Δ::*λimm434*/*ura3*Δ::*λimm434*, *his1*Δ::*hisG*/*HIS1*, *arg4*Δ::*hisG*/*arg4*Δ::*hisG*	Lab collection
BWP17AH	CEC161	BWP17	*ura3*Δ::*λimm434*/*ura3*Δ::*λimm434*, *his1*Δ::*hisG*/*HIS1*, *arg4*Δ::*hisG*/*ARG4*	[86]
SN76	CEC805		*arg4*Δ/*arg4*Δ, *his1*Δ/*his1*Δ, *ura3*Δ::*λimm434*/*ura3*Δ::*λimm434*, *iro1*Δ::*λimm434*/*iro1*Δ::*λimm434*	[87]
HLC52	CEC150		*ura3*Δ::*λimm434*/*ura3*Δ::*λimm434*, *efg1*Δ::*hisG*/*efg1*Δ::*hisG*-*URA3*-*hisG*	[17]
HLCEEFG1	CEC3891	CAI4	*ura3*Δ::*λimm434*/*ura3*Δ::*λimm434*, *efg1::hisG/efg1::EFG1-HA-URA3*	[18]
AVL12	CEC3894	BWP17	*ura3Δ::λimm434/ura3Δ::λimm434, arg4Δ::hisG/arg4Δ::hisG, his1Δ::hisG/his1Δ::hisG, efg1::hisG/efg1::EFG1-HA-URA3*	[18]
AVL12-SFL1-TAP	CEC3923	AVL12	*ura3Δ::λimm434/ura3Δ::λimm434, arg4Δ::hisG/arg4Δ::hisG, his1Δ::hisG/his1Δ::hisG, efg1::hisG/efg1::EFG1-HA-URA3, SFL1/SFL1-TAP-HIS1*	This study
AVL12-SFL2-TAP	CEC3916	AVL12	*ura3Δ::λimm434/ura3Δ::λimm434, arg4Δ::hisG/arg4Δ::hisG, his1Δ::hisG/his1Δ::hisG, efg1::hisG/efg1::EFG1-HA-URA3, SFL2/SFL2-TAP-HIS1*	This study
AVL12-pHIS	CEC3913	AVL12	*ura3Δ::λimm434/ura3Δ::λimm434, arg4Δ::hisG/arg4Δ::hisG, his1Δ::HIS1/his1Δ::hisG, efg1::hisG/efg1::EFG1-HA-URA3*	This study
SGY243-CaEXP-B	CEC2894	SGY243	*RPS1*::(pCaEXP) *URA3* P*_MET3_*	[43]
SGY243-CaEXP-CAP1-HA	CEC2895	SGY243	*RPS1*::(pCaEXP) *URA3* P*_MET3_*-*CAP1-HA_3_*	[43]
CEC1561	CEC1561	SN76	*sfl1*Δ::*ARG4/SFL1*	This study
SFL1-TAP	CEC1922	CEC1561	*sfl1Δ::ARG4/SFL1-TAP-HIS1 RPS1/RPS1::*(CIp10) *URA3*	This study
CEC1422	CEC1422	SN76	*sfl2*Δ::*ARG4/SFL2*	This study
SFL2-TAP	CEC1918	CEC1422	*sfl1Δ::ARG4/SFL2-TAP-HIS1 RPS1/RPS1::*(CIp10) *URA3*	This study
CEC3075	CEC3075	CEC1561	*sfl1*Δ::*ARG4/sfl1*::*SFL1-HA_3_*-*URA3*-*HA_3_*	This study
CEC3076	CEC3076	CEC1422	*sfl2Δ::ARG4/sfl2::SFL2-HA_3_*-*URA3*-*HA_3_*	This study
*sfl1*Δ/*sfl1*Δ	CEC1910	CEC1561	*sfl1*Δ::*ARG4*/*sfl1*Δ::*HIS1*	This study
CEC1997	CEC1997	CEC1910	*sfl1δ::ARG4/sfl1Δ::HIS1*, *RPS1/rps1::*(CIp10) *URA3 P_PCK1_-SFL1-TAP*	This study
*sfl1*-CaEXP	CEC3283	CEC1910	*sfl1*Δ::*ARG4*/*sfl1*Δ::*HIS1*, *RPS1*/*RPS1*::(pCaEXP) *URA3* P*_MET3_*	This study
*sfl1*-CaEXP-*SFL1-HA_3_*	CEC3284	CEC1910	*sfl1*Δ::*ARG4*/*sfl1*Δ::*HIS1*, *RPS1*/*RPS1*::(pCaEXP) *URA3* P*_MET3_*-*SFL1-HA_3_*	This study
*sfl2*Δ/*sfl2*Δ	CEC1503	CEC1422	*sfl2*Δ::*ARG4*/*sfl2*Δ::*HIS1*	This study
*sfl2*-CaEXP	CEC3253	CEC1503	*sfl2*Δ::*ARG4*/*sfl2*Δ::*HIS1*, *RPS1*/*RPS1*::(pCaEXP) *URA3* P*_MET3_*	This study
*sfl2*-CaEXP-*SFL2-HA_3_*	CEC3254	CEC1503	*sfl2*Δ::*ARG4*/*sfl2*Δ::*HIS1*, *RPS1*/*RPS1*::(pCaEXP) *URA3* P*_MET3_*-*SFL2-HA_3_*	This study
*sfl1*ΔΔ	CEC2001	CEC1910	*sfl1*Δ::*ARG4*/*sfl1*Δ::*HIS1*, *RPS1*/*RPS1*::(CIp10) *URA3*	This study
*sfl1*ΔΔ *sfl2*ΔΔ	CEC2658	CEC1910	*sfl1*Δ::*ARG4*/*sfl1*Δ::*HIS1*, *sfl2*Δ::*URA3*/*sfl2*Δ::*SAT1*	This study
*sfl2*ΔΔ	CEC1535	CEC1503	*sfl2*Δ::*ARG4*/*sfl2*Δ::*HIS1*, *RPS1*/*RPS1*::(CIp10) *URA3*	This study
CEC1509	CEC1509	CEC1503	*sfl2Δ::ARG4/sfl2Δ::HIS1*, *RPS1/rps1::*(CIp10) *URA3 P_PCK1_-SFL2-TAP*	This study
*sfl1*ΔΔ *brg1*ΔΔ	CEC2840	CEC1910	*sfl1*Δ::*ARG4*/*sfl1*Δ::*HIS1*, *brg1*Δ::*URA3*/*brg1*Δ::*SAT1*	This study
*brg1*Δ/*brg1*Δ	CEC2009	SN76	*brg1*Δ::*ARG4*/*brg1*Δ::*HIS1*	This study
*brg1*ΔΔ	CEC2058	CEC2009	*brg1*Δ::*ARG4*/*brg1*Δ::*HIS1*, *RPS1*/*RPS1*::(CIp10) *URA3*	This study
CEC3485	CEC3485	BWP17AH	*ADH1*/*adh1*::P*_TDH3_*-*carTA*::*SAT1*, *RPS1*/*RPS1*::(CIp10) *URA3*	This study
CEC2988	CEC2988	BWP17AH	*ADH1*/*adh1*::P*_TDH3_*-*carTA*::*SAT1*, *RPS1*/*RPS1*::(CIp10) *URA3* P*_TET_*-*SFL2*	This study
CEC3431	CEC3431	CEC1910	*sfl1*Δ::*ARG4*/*sfl1*Δ::*HIS1*, *ADH1*/*adh1*::P*_TDH3_*-*carTA*::*SAT1*, *RPS1*/*RPS1*::(CIp10) *URA3*	This study
CEC3484	CEC3484	CEC1910	*sfl1*Δ*::ARG4/sfl1*Δ*::HIS1*, *ADH1*/*adh1*::P*_TDH3_*-*carTA*::*SAT1*, *RPS1*/*RPS1*::(CIp10) *URA3* P*_TET_*-*SFL2*	This study
CEC3435	CEC3435	CEC1503	*sfl2*Δ::*ARG4*/*sfl2*Δ::*HIS1*, *ADH1*/*adh1*::P*_TDH3_*-*carTA*::*SAT1*, *RPS1*/*RPS1*::(CIp10) *URA3*	This study
CEC3437	CEC3437	CEC1503	*sfl2*Δ::*ARG4*/*sfl2*Δ::*HIS1*, *ADH1*/*adh1*::P*_TDH3_*-*carTA*::*SAT1*, *RPS1*/*RPS1*::(CIp10) *URA3* P*_TET_*-*SFL2*	This study
*ume6*Δ/*ume6*Δ	CEC2656	SN76	*ume6*Δ::*ARG4*/*ume6*Δ::*HIS1*	This study
CEC3583	CEC3583	CEC2656	*ume6*Δ::*ARG4*/*ume6*Δ::*HIS1*, *ADH1*/*adh1*::P*_TDH3_*-*carTA*::*SAT1*, *RPS1*/*RPS1*::(CIp10) *URA3*	This study
CEC3585	CEC3585	CEC2656	*ume6*Δ::*ARG4*/*ume6*Δ::*HIS1*, *ADH1*/*adh1*::P*_TDH3_*-*carTA*::*SAT1*, *RPS1*/*RPS1*::(CIp10) *URA3* P*_TET_*-*SFL2*	This study
*tec1*Δ/*tec1*Δ	CEC2335	SN76	*tec1*Δ::*ARG4*/*tec1*Δ::*HIS1*	This study
CEC3589	CEC3589	CEC2335	*tec1*Δ::*ARG4*/*tec1*Δ::*HIS1*, *ADH1*/*adh1*::P*_TDH3_*-*carTA*::*SAT1*, *RPS1*/*RPS1*::(CIp10) *URA3*	This study
CEC3591	CEC3591	CEC2335	*tec1*Δ::*ARG4*/*tec1*Δ::*HIS1*, *ADH1*/*adh1*::P*_TDH3_*-*carTA*::*SAT1*, *RPS1*/*RPS1*::(CIp10) *URA3* P*_TET_*-*SFL2*	This study
CEC3581	CEC3581	CEC2009	*brg1*Δ::*ARG4*/*brg1*Δ::*HIS1*, *ADH1*/*adh1*::P*_TDH3_*-*carTA*::*SAT1*, *RPS1*/*RPS1*::(CIp10) *URA3*	This study
CEC3642	CEC3642	CEC2009	*brg1*Δ::*ARG4*/*brg1*Δ::*HIS1*, *ADH1*/*adh1*::P*_TDH3_*-*carTA*::*SAT1*, *RPS1*/*RPS1*::(CIp10) *URA3* P*_TET_*-*SFL2*	This study
*efg1*Δ/*efg1*Δ	CEC1439	HLC52	*ura3*Δ::*λimm434*/*ura3*Δ::*λimm434*, *efg1*Δ::*hisG*/*efg1*Δ::*hisG*	This study
CEC3581	CEC3581	CEC1439	*efg1*Δ::*ARG4*/*efg1*Δ::*HIS1*, *ADH1*/*adh1*::P*_TDH3_*-*carTA*::*SAT1*, *RPS1*/*RPS1*::(CIp10) *URA3*	This study
CEC3156	CEC3156	CEC1439	*efg1*Δ::*ARG4*/*efg1*Δ::*HIS1*, *ADH1*/*adh1*::P*_TDH3_*-*carTA*::*SAT1*, *RPS1*/*RPS1*::(CIp10) *URA3* P*_TET_*-*SFL2*	This study

To test the functionality of the Sfl1p-HA_3_ and Sfl2p-HA_3_ fusions, both tagged and empty-vector control strains were grown overnight at 30°C in YPD then transferred to Lee's medium (hyphae-inducing medium) lacking methionine (P*_MET3_*-inducing condition) at 37°C and allowed to resume growth during 4 h prior to microscopic examination (Figure 1C). It was previously shown that P*_MET3_*-driven expression of wild-type *SFL1* in a homozygous *sfl1* mutant strain under hyphae-inducing conditions abolished hyphal formation [37]. As expected, hyphal formation was induced in either the control strain SC5314 or the *sfl1*Δ/*sfl1*Δ mutant carrying the empty vector (Figure 1C, top left and middle panels, respectively). Conversely, hyphal formation was strongly impaired in the strain expressing *SFL1-HA_3_* (Figure 1C, top right panel), therefore phenocopying the effect of P*_MET3_*-driven wild-type *SFL1* expression as observed in Bauer *et al.* [37]. Under the same growth conditions the *sfl2*Δ/*sfl2*Δ strain carrying the empty vector was unable to form hyphae (Figure 1C, bottom middle panel), whereas expression of the *SFL2-HA_3_* allele allowed induction of hyphal formation as observed in strain SC5314 (Figure 1, compare bottom left and right panels). Taken together, these results show that epitope-tagging of Sfl1p and Sfl2p at their C-termini using the pCaEXP system allowed the production of fully functional proteins.

### Genome-wide location map of Sfl1p and Sfl2p at a single nucleotide resolution

We performed genome-wide location of Sfl1p or Sfl2p under hyphae-inducing conditions by chromatin immunoprecipitation coupled to massively parallel high-throughput sequencing (ChIP-Seq, see Materials and Methods), which allows to detect binding events at a single nucleotide resolution. The resulting reads were mapped to the *C. albicans* Assembly 21 genome and alignments were visualized using the Integrative Genomics Viewer (IGV) software [44], [45] (see Materials and Methods for details). Using the Model-Based Analysis for ChIP-Seq (MACS) peak-finding algorithm [46], we identified 163 and 213 binding peaks for Sfl1p and Sfl2p, respectively (see Tables S1–S6 in Text S1, Legends to Supplementary Tables S1–S8 in Text S1 and Materials and Methods for details).

**Figure 2:**
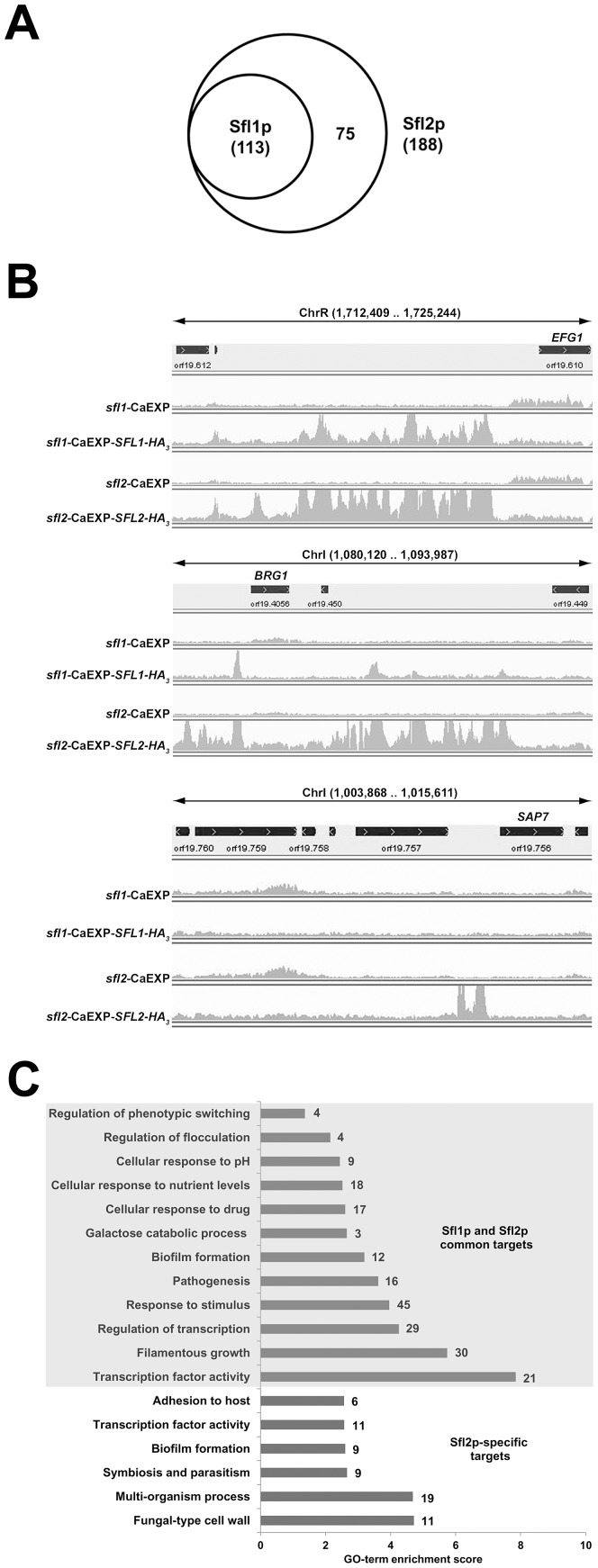
Genome-wide location of *Candida albicans* Sfl1p and Sfl2p, *in vivo*, at a single-nucleotide resolution. (**A**) Venn diagram of the overlap between Sfl1p and Sfl2p binding targets. All 113 Sfl1p targets are also bound by Sfl2p, while 75 target promoters are Sfl2p-specific. The total number of Sfl1p or Sfl2p target promoters are indicated between parentheses. Target promoters include those that are clearly associated with given ORFs as well as those that are shared by two ORFs in opposite orientations. (**B**) A single-nucleotide resolution of Sfl1p and Sfl2p binding at selected *C. albicans* genomic regions *in vivo*. Plotted are read-count signal intensities of HA_3_-tagged *SFL1*- (*sfl1*-CaEXP-*SFL1-HA_3_*) or *SFL2*- (*sfl2*-CaEXP-*SFL2-HA_3_*) coimmunoprecipitated DNA and the corresponding empty-vector control signals (*sfl1*-CaEXP, *sfl2*-CaEXP, respectively) from merged BAM files of two independent biological replicates. Some read-count signals extend beyond the maximum graduation (not shown) that ranges between 0–500 reads for Sfl1 data (*sfl1*-CaEXP and *sfl1*-CaEXP-*SFL1-HA_3_*) and 0–1000 reads for Sfl2 data (*sfl2*-CaEXP and *sfl2*-CaEXP-*SFL2-HA_3_*). The position of each signal in selected *C. albicans* genomic regions from assembly 21 is shown on the *x*-axis. The location of each selected region from the corresponding chromosome (Chr) is indicated at the top of each panel (limits are shown between parentheses in base pairs). The orientation of each ORF is depicted by the arrowed black rectangle. (**C**) Enrichment scores of the Gene Ontology (GO) terms to which are assigned Sfl1p and Sfl2p common (shaded area) or Sfl2p-specific (unshaded area) binding targets. GO term enrichment scores are calculated as the negative value of the log_10_-transformed *P*-value. The number of genes of each category is shown at the right of each horizontal bar.

As expected, most of Sfl1p or Sfl2p binding peaks were located at ‘intergenic’ regions (Tables S1–S6 in Text S1), consistent with a transcriptional regulatory function. Among the 163 Sfl1p binding peaks, 76 clearly associated with individual ORFs, while 34 were located at promoter regions shared by two ORFs in opposite orientations and the remaining 53 peaks were not clearly associated with ORFs. In particular, spurious binding overlapping with highly transcribed regions [47], mostly tRNA-encoding genes, or regions with repeated DNA sequence (Table S3 in Text S1), was observed. Among the 213 Sfl2p binding peaks, 140 clearly associated with unique ORFs, while 54 were located in promoter regions shared by two ORFs in opposite orientations and the remaining 19 peaks were not clearly linked to defined ORFs (Table S6 in Text S1). Additional *bona fide* Sfl1p (14 peaks) and Sfl2p (28 peaks) binding peaks were not detected by the peak-finding algorithm and were added to our target lists (Tables S3 and S6 in Text S1, see column entitled “comments” and Legends to Supplementary Tables S1–S8 in Text S1). Overall, examination of Sfl1p and Sfl2p binding peaks allowed to identify 113 and 188 target promoters (Figure 1A) including 39 and 56 promoter regions shared by two ORFs, respectively. Interestingly, all 113 Sfl1p targets were also bound by Sfl2p, suggesting functional interactions between the two regulators, while 75 additional targets were specific to Sfl2p (Figure 2A). In many occurrences, Sfl2p binding at promoter regions strongly overlapped with that of Sfl1p (Figure 2B, top panel as an example). In other cases, Sfl2p binding showed partial (Figure 2B, middle panel as an example) or no overlap (Figure 2B, bottom panel as an example) with Sfl1p binding. Noteworthy, Sfl2p and Sfl1p binding peaks were often lying across relatively long regions, particularly in the vicinity of transcription factor-encoding genes such as *EFG1* (Figure 2B, top panel), *UME6*, *NRG1* or *TEC1*, suggesting the presence of more than one binding site or the existence of functional interactions with other regulatory proteins at these sites.

We used the GO Term Finder tool from the CGD [48] to identify functional enrichment among Sfl1p and Sfl2p targets relative to the annotated *C. albicans* genome (Table 2; see Materials and Methods). Strikingly, we found that the most significantly enriched functional category among Sfl1p and Sfl2p common targets was “Sequence-specific DNA-binding transcription activity” (21 genes, *P* = 1.42×10^−8^; Figure 2C, grey shading), including a large number of genes encoding major transcription factors involved in *C. albicans* morphogenesis and virulence such as *UME6*, *TEC1*, *NRG1*, *RFG1*, *BRG1*, *FLO8*, others (Figure 2C and Table 2). In line with this finding, the functional grouping “Filamentous growth” (30 genes, *P* = 1.83×10^−6^) was also among the most overrepresented categories of the identified GO terms and included the above-mentioned transcription factors in addition to *HMS1*, encoding a transcription factor that controls *C. albicans* morphogenesis mediated by *HSP90* compromise or high temperature [49], as well as many genes encoding effectors or signal transducers of this process such as *MSB2*, *CHT2*, *GAP1*, *ALS1*, *RAS2*, others (Figure 2C). As expected, “Pathogenesis” (16 genes; *P* = 2.40×10^−4^) was also among the most significantly enriched functional categories among Sfl1p and Sfl2p common targets and is consistent with the known roles of Sfl1p and Sfl2p in *C. albicans* virulence [38], [39]. Interestingly, Sfl1p and Sfl2p bound to genes encoding transcription factors involved in white/opaque switching, including *WOR2*, *FLO8*, *EFG1* and *AHR1* (“Regulation of phenotypic switching”; 4 genes; *P* = 4.34×10^−2^), as well as genes involved in biofilm formation (“Biofilm formation”; 12 genes; *P* = 6.40×10^−4^), suggesting wider functions for these two regulators in *C. albicans*. These functions may include the ability to respond to a variety of stimuli, such as drug treatment (“Cellular response to drug”; 17 genes; *P* = 2.48×10^−3^), nutrient availability (“Cellular response to nutrient levels”; 18 genes; *P* = 3.00×10^−3^ and “Galactose catabolic process via UDP-Galactose”; 3 genes; P = 2.23×10^−3^) and pH levels (“Cellular response to pH; 9 genes; *P* = 3.62×10^−3^).

**Table 2:** Overrepresented functional categories in Sfl1p and Sfl2p ChIP-Seq data.

GO term^a^	CGD accession # (ontology classification)^b^	% Frequency^c^ (# of genes)	% Genome frequency^d^ (# of genes)	*P* value^e^	Genes^f^
**Sfl1p and Sfl2p common targets**					
Sequence-specific DNA binding transcription factor activity	GO:0003700	19.1 (21)	3.5 (230)	0.0000	*FLO8*, *UME6*, *FGR15*, *CRZ2*, *RFG1*, *SEF1*, *SFL2*, *BRG1*, *MIG1*, *RME1*, *STP2*, *TEC1*, *ZCF31*, *WOR2*, *EFG1*, *CUP9*, *FCR1*, *NRG1*, *BCR1*, *CTA4*, *AHR1*
Filamentous growth	GO:0030447	27.3 (30)	8.4 (550)	0.0000	*FLO8*, *MSB2*, *UME6*, *FGR15*, *RFG1*, *GAL10*, *SEF1*, *CHT2*, *SFL2*, *BRG1*, *GAP1*, orf19.4459, *STP2*, *ALS1*, *RAS2*, *TEC1*, *WOR2*, *RHB1*, *EFG1*, *CUP9*, *TCC1*, *SSN6*, *FCR1*, orf19.6874, *NRG1*, *BCR1*, *CTA4*, *AHR1*, *AAF1*, *HMS1*
Regulation of transcription, DNA-dependent	GO:0006355	26.4 (29)	9.2 (601)	0.0001	*FLO8*, *UME6*, *FGR15*, *CRZ2*, *RFG1*, *GAL1*, *SEF1*, *SFL2*, *CTA24*, *BRG1*, *MIG1*, *RME1*, *STP2*, *TEC1*, *ZCF31*, *WOR2*, *EFG1*, *CUP9*, *TCC1*, *SSN6*, *FCR1*, orf19.6874, *NRG1*, *BCR1*, *CTA4*, *AHR1*, *HAP41*, *AAF1*, *HMS1*
Response to stimulus	GO:0050896	40.9 (45)	19.8 (1290)	0.0001	*FLO8*, *MSB2*, *UME6*, *HNM1*, *REG1*, *FGR15*, *SIT1*, *CRZ2*, orf19.2726, orf19.2822, *RFG1*, *GSC1*, *DIP5*, *GAL1*, *GAL10*, *GAL102*, *SEF1*, *CHT2*, *SFL2*, *BRG1*, *FET3*, *FET34*, *MIG1*, orf19.4459, *SWE1*, orf19.4883, *STP2*, *MDR1*, *ALS1*, *RAS2*, *TEC1*, *ZCF31*, *RHB1*, *EFG1*, *HSP104*, *TCC1*, *SSN6*, *FCR1*, *GAC1*, *NRG1*, *BCR1*, *CTA4*, *AHR1*, *GPX2*, *HMS1*
Pathogenesis	GO:0009405	14.5 (16)	3.3 (215)	0.0002	*FLO8*, *UME6*, *RFG1*, *GSC1*, *SFL2*, *BRG1*, *SWE1*, *MDR1*, *ALS1*, *TEC1*, *EFG1*, *HSP104*, *TCC1*, *SSN6*, *NRG1*, *AHR1*
Biofilm formation	GO:0042710	10.9 (12)	2.0 (128)	0.0006	*FLO8*, *CRZ2*, *YWP1*, *BRG1*, *ALS1*, *TEC1*, *ZCF31*, *EFG1*, *HSP104*, *NRG1*, *BCR1*, *AHR1*
Galactose catabolic process via UDP-galactose	GO:0033499	2.7 (3)	0.0 (3)	0.0022	*GAL1*, *GAL10*, *GAL7*
Cellular response to drug	GO:0035690	15.5 (17)	4.4 (287)	0.0025	*HNM1*, *SIT1*, *GSC1*, *DIP5*, *GAL102*, *FET3*, *MIG1*, *SWE1*, *STP2*, *MDR1*, *ZCF31*, *RHB1*, *EFG1*, *SSN6*, *FCR1*, *NRG1*, *AHR1*
Cellular response to nutrient levels	GO:0031669	16.4 (18)	5.0 (323)	0.0030	*UME6*, *REG1*, *FGR15*, orf19.2822, *RFG1*, *GAL1*, *GAL10*, *CHT2*, *BRG1*, *MIG1*, orf19.4459, *RAS2*, *RHB1*, *EFG1*, *FCR1*, *NRG1*, *BCR1*, *AHR1*
Cellular response to pH	GO:0071467	8.2 (9)	1.2 (81)	0.0036	*UME6*, *CRZ2*, *SEF1*, *SFL2*, *STP2*, *ALS1*, *EFG1*, *TCC1*, *NRG1*
Regulation of flocculation	GO:0060256	3.6 (4)	0.2 (10)	0.0071	*FLO8*, *GAL10*, *SFL2*, *ALS1*
Regulation of phenotypic switching	GO:1900239	3.6 (4)	0.2 (15)	0.0434	*FLO8*, *WOR2*, *EFG1*, *AHR1*
**Sfl2p-specific targets**					
Fungal-type cell wall	GO:0009277	15.1 (11)	2.2 (142)	0.0000	*HWP1*, *EAP1*, *ALS3*, *PIR1*, *HYR1*, *SIM1*, *RBR3*, *PGA31*, *RHD3*, *WSC1*, *ALS6*
Multi-organism process	GO:0051704	26.0 (19)	6.4 (418)	0.0000	*CPH2*, *HWP1*, *EAP1*, *ALS3*, *CZF1*, *FCR3*, *ECE1*, *SFL1*, *RFX2*, *HYR1*, *ROB1*, *RHD3*, *SAP4*, *SRR1*, *ADE2*, *HGC1*, *RBT4*, *ALS6*, *SAP7*
Symbiosis, encompassing mutualism through parasitism	GO:0044403	12.3 (9)	1.9 (126)	0.0022	*CPH2*, *HWP1*, *EAP1*, *ALS3*, *RFX2*, *HYR1*, *SAP4*, *HGC1*, *ALS6*
Biofilm formation	GO:0042710	12.3 (9)	2.0 (128)	0.0025	*HWP1*, *EAP1*, *ALS3*, *CZF1*, *FCR3*, *ECE1*, *HYR1*, *ROB1*, *ALS6*
Sequence-specific DNA binding transcription factor activity	GO:0003700	15.1 (11)	3.5 (230)	0.0027	*CPH2*, orf19.1604, *ECM22*, *CZF1*, *FCR3*, orf19.3328, *GRF10*, orf19.4342, *SFL1*, *RFX2*, *ROB1*
Adhesion to host	GO:0044406	8.2 (6)	0.7 (47)	0.0027	*HWP1*, *EAP1*, *ALS3*, *RFX2*, *HYR1*, *SAP4*

a Grouping of the Sfl1p and/or Sfl2p targets identified in ChIP-Seq data according to GO terminology determined by using the online CGD GO Term Finder tool (http://www.candidagenome.org/cgi-bin/GO/goTermFinder). Analysis conducted in October 2012 (See Materials and Methods).

b Ontology classification according to the three GO terminologies (biological process, cellular component and molecular function).

c Percentages were calculated based on the number of genes in each GO category divided by the total number (110 genes for Sfl1p and Sfl2p common targets, 73 genes for Sfl2p specific targets, see Materials and Methods for details).

d Percentages were calculated based on the number of genes in each category divided by the total number of annotated genes of the *C. albicans* genome, according to CGD (6,513 genes).

e *P* values for the overrepresented categories were calculated using a hypergeometric distribution with multiple hypothesis correction (i.e., Bonferroni's correction) as described in the GO Term Finder tool website (http://www.candidagenome.org/help/goTermFinder.shtml). The *P* value cutoff used was ≤0.05.

f Gene name or orf19 nomenclature according to CGD. Some genes were attributed to more than one GO term.

We also performed functional category enrichment analyses of the 75 Sfl2p-specific targets (Figure 2C, unshaded area). Interestingly, these targets were grouped into functional categories pertaining to interaction with the host, including “Multi-organism process” (19 genes; *P* = 2.06×10^−5^), “Symbiosis, encompassing mutualism through parasitism” (9 genes; *P* = 2.18×10^−3^), “Adhesion to host” (6 genes; *P* = 2.69×10^−3^) and “Fungal-type cell wall” (11 genes; *P* = 1.92×10^−5^). Sfl2p also bound specifically to 11 genes encoding transcription factors such as *CPH2*, *ECM22*, *CZF1*, *FCR3*, *RFX2* and *ROB1* (Table 2). We also found that Sfl2p bound specifically to the *SFL1* promoter, while both Sfl1p and Sfl2p bound to the promoter of *SFL2*, suggesting an autoregulatory loop controlling *SFL2* expression.

**Figure 3:**
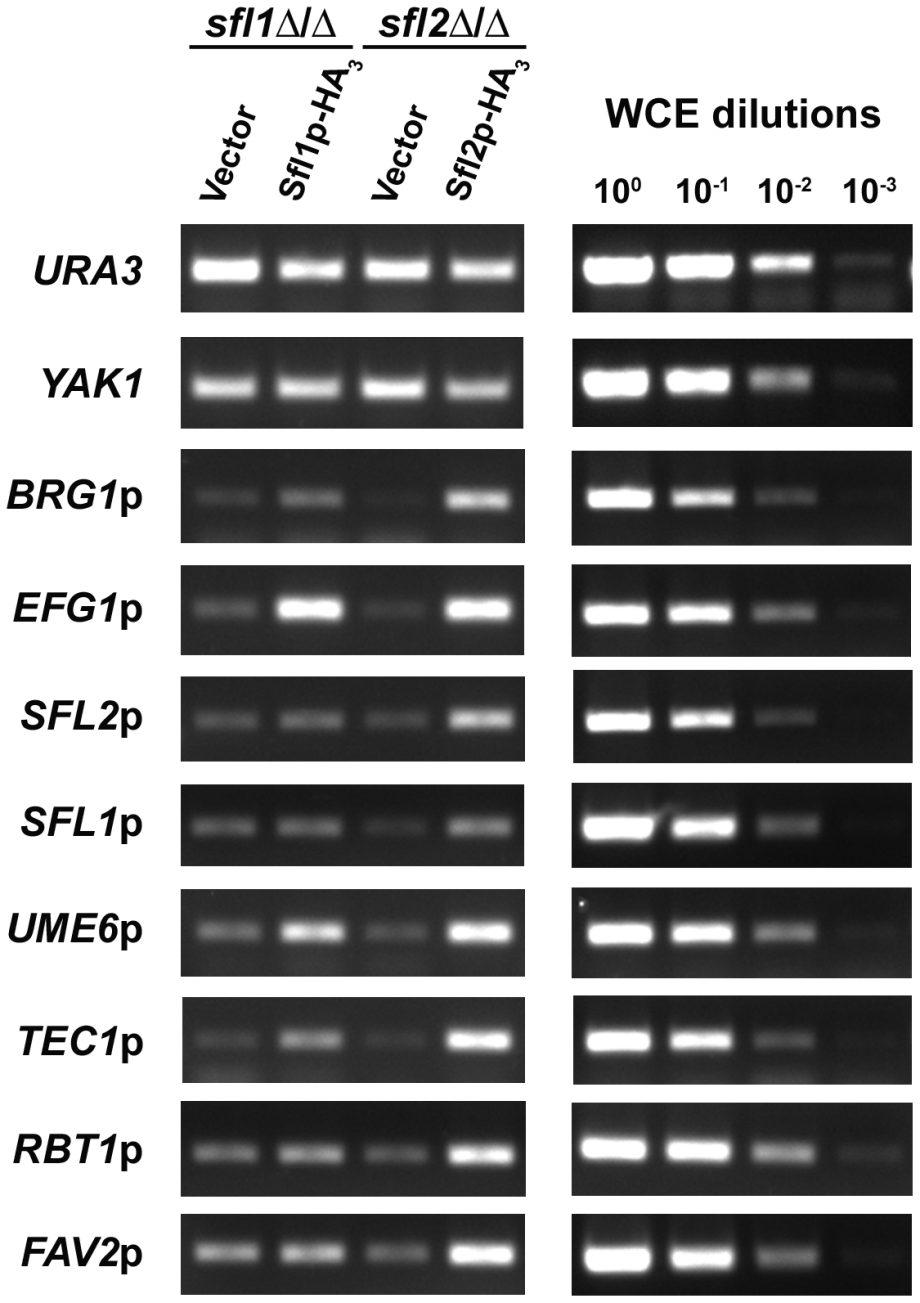
Binding of Sfl1p-HA_3_ and Sfl2p-HA_3_ to selected target promoters. Strains *sfl1*-CaEXP-*SFL1-HA_3_* (Sfl1p-HA_3_) and *sfl2*-CaEXP-*SFL2-HA_3_* (Sfl2p-HA_3_) together with their respective untagged control strains (Vector) were grown under the same conditions as those for the ChIP-Seq experiment prior to ChIP followed by PCR to detect specific Sfl1p and Sfl2p binding enrichment at selected target promoters (See Materials and Methods for details). PCR was performed using primers corresponding to the promoter region of the indicated genes. The *URA3* and *YAK1* genes were used as a negative control for ChIP enrichment. Primer efficiency (shown on the right panel) was tested by the ability of the corresponding primers to quantify 10-fold serially diluted whole cell extract DNA (WCE, ChIP input samples, dilution factors are indicated at the top of the right panel).

To validate our ChIP-Seq data, we performed additional independent ChIP experiments and measured Sfl1p and Sfl2p binding by PCR (ChIP-PCR) on selected targets (Figure 3). The *URA3* and *YAK1* genes were used as negative controls for ChIP enrichment. As expected, Sfl1p and Sfl2p binding was detected at the promoter of their targets, including *BRG1*, *EFG1*, *SFL2*, *UME6* and *TEC1* (Figure 3). The promoter region of Sfl2p-specific targets was also enriched by Sfl2p-HA_3_ immunoprecipitation, including *SFL1*, *RBT1* and *FAV2*, but not by the immunoprecipitation of Sfl1p-HA_3_ (Figure 3).

Taken together, our results suggest that Sfl1p and Sfl2p regulate *C. albicans* morphogenesis and potentially confer virulence through direct binding to the promoter of genes encoding key regulators of these processes. They also revealed that, while both transcription factors bind to common targets, Sfl2p specifically binds to additional target genes that appear to be involved in processes pertaining to interaction with the host.

### Global gene expression profiling reflects the antagonistic functions of *SFL1* and *SFL2* in regulating *C. albicans* morphogenesis

**Figure 4:**
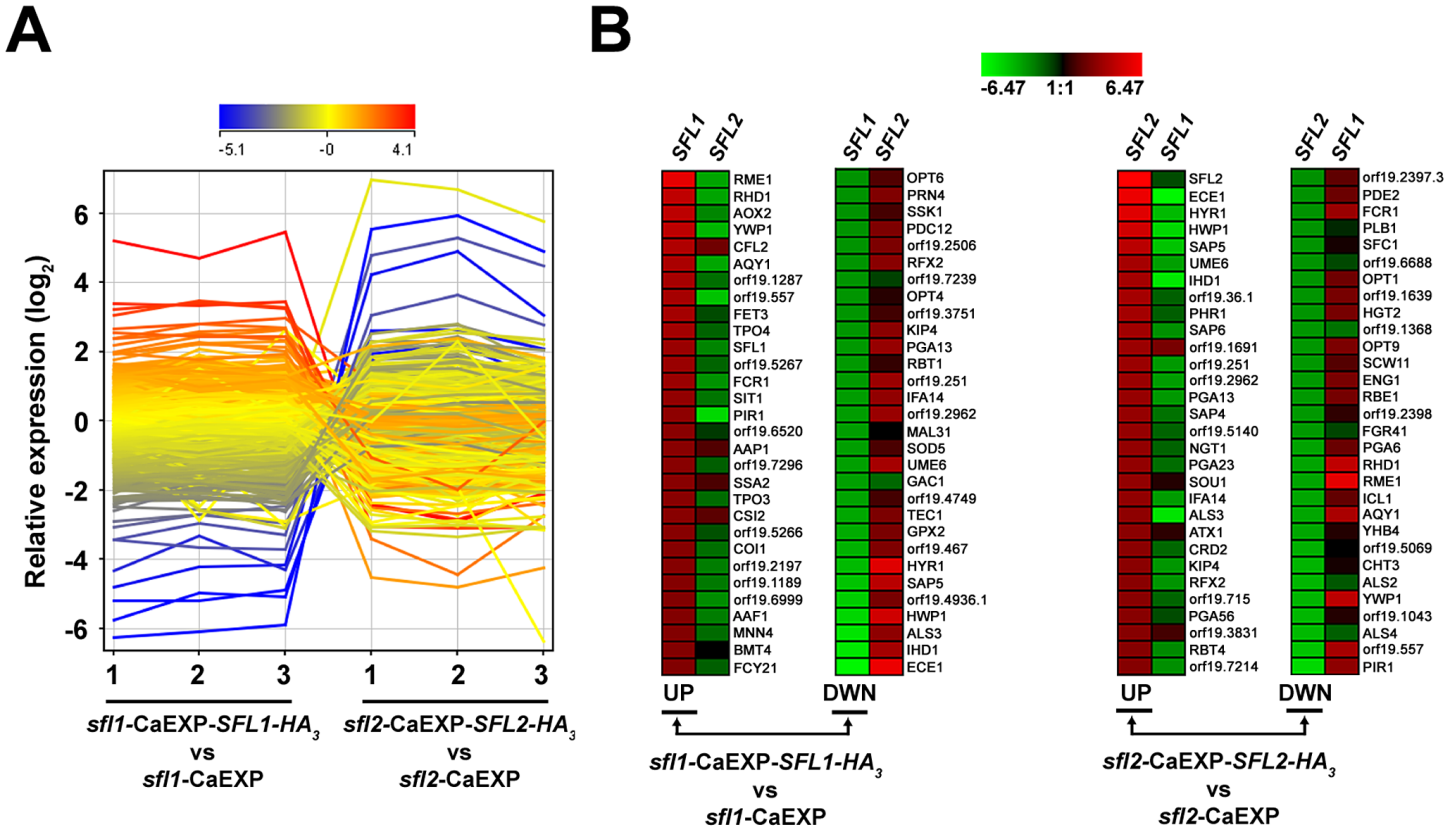
Sfl1p and Sfl2p transcriptomics. (**A**) GeneSpring expression profile plots of each of the three biological replicates from the *sfl1*-CaEXP-*SFL1-HA_3_* versus *sfl1*-CaEXP (*sfl1*-CaEXP-*SFL1-HA_3_* vs. *sfl1*-CaEXP) and the *sfl2*-CaEXP-*SFL2-HA_3_* versus *sfl2*-CaEXP (*sfl2*-CaEXP-*SFL2-HA_3_* vs. *sfl2*-CaEXP) transcriptomics data. The log_2_-transformed relative expression level of each gene from averaged signal intensities of two nonoverlapping gene-specific microarray probes (See Materials and Methods for details), is shown on the *y*-axis and the corresponding biological replicate sample for each condition (1, 2 and 3) is shown on the *x*-axis. The profile plot is coloured according to the ratio observed for replicate 1 in the *sfl1*-CaEXP-*SFL1-HA_3_* vs. *sfl1*-CaEXP condition. (**B**) Heat maps of the 30 highest log_2_-transformed relative gene expression levels in the *sfl1*-CaEXP-*SFL1-HA_3_* versus *sfl1*-CaEXP (*sfl1*-CaEXP-*SFL1-HA_3_* vs *sfl1*-CaEXP, left panels, UP and DWN) and the *sfl2*-CaEXP-*SFL2-HA_3_* versus *sfl2*-CaEXP (*sfl2*-CaEXP-*SFL2-HA_3_* vs *sfl2*-CaEXP, right panels, UP and DWN) transcriptomics data (combination of the 3 biological replicates in each condition). The most upregulated (UP, descending signal intensity) or downregulated (DWN, ascending signal intensity) genes in *sfl1*-CaEXP-*SFL1-HA_3_* vs. *sfl1*-CaEXP (left panels, *SFL1* column) or *sfl2*-CaEXP-*SFL2-HA_3_* vs. *sfl2*-CaEXP (*SFL2*, right panels) transcriptomics data and their matching probe intensities from the *sfl2*-CaEXP-*SFL2-HA_3_* vs. *sfl2*-CaEXP condition (left panels, *SFL2* column) or the *sfl1*-CaEXP-*SFL1-HA_3_* vs. *sfl1*-CaEXP (right panels, *SFL1* column), respectively, are indicated with their corresponding name or orf19 nomenclature. Heat maps were constructed using Genesis version 1.7.6 [83].

To determine whether Sfl1p and Sfl2p binding targets were also transcriptionally modulated, we performed global gene expression analyses of strains *sfl1*-CaEXP-*SFL1-HA_3_* versus *sfl1*-CaEXP and *sfl2*-CaEXP-*SFL2-HA_3_* versus *sfl2*-CaEXP grown 3 times independently under the same conditions than those in the ChIP-Seq experiments (see Materials and Methods for details). We found 643 upregulated and 579 downregulated genes (expression fold-change ≥1.5; *P*≤0.05) in the *sfl1*-CaEXP-*SFL1-HA_3_* strain as compared to strain *sfl1*-CaEXP (Table S7 in Text S1). On the other hand, 354 genes were upregulated and 478 genes were downregulated (expression fold-change ≥1.5; *P*≤0.05) in strain *sfl2*-CaEXP-*SFL2-HA_3_* relative to *sfl2*-CaEXP (Table S8 in Text S1). Data were visualized using an expression profile plot (GeneSpring version 12, Agilent Technologies), which allows to get a global view of gene expression variation and thus to compare the expression patterns in *SFL1* and *SFL2* data sets (Figure 4A). Interestingly, most of the highly upregulated genes in pCaEXP-*SFL1-HA_3_* vs. pCaEXP data were strongly downregulated in pCaEXP-*SFL2-HA_3_* vs. pCaEXP data (Figures 4A and 4B left panel). Many of these genes are markers of the yeast form growth phase, such as *RME1*, *YWP1*, *RHD1* and orf19.557. On the other hand, most of the strongly downregulated genes in pCaEXP-*SFL1-HA_3_* vs. pCaEXP data were actually upregulated in pCaEXP-*SFL2-HA_3_* vs. pCaEXP data (Figure 4A), including the HSGs *ECE1*, *ALS3*, *IHD1*, *HWP1*, *HYR1* and *SAP5* (Figure 4B). Examination of the genes that were strongly modulated in pCaEXP-*SFL2-HA_3_* vs. pCaEXP data also revealed similar gene expression dynamics: many of the upregulated genes were found to be downregulated in pCaEXP-*SFL1-HA_3_* vs. pCaEXP data sets, and *vice versa* (Figure 4B, right panel).

**Figure 5:**
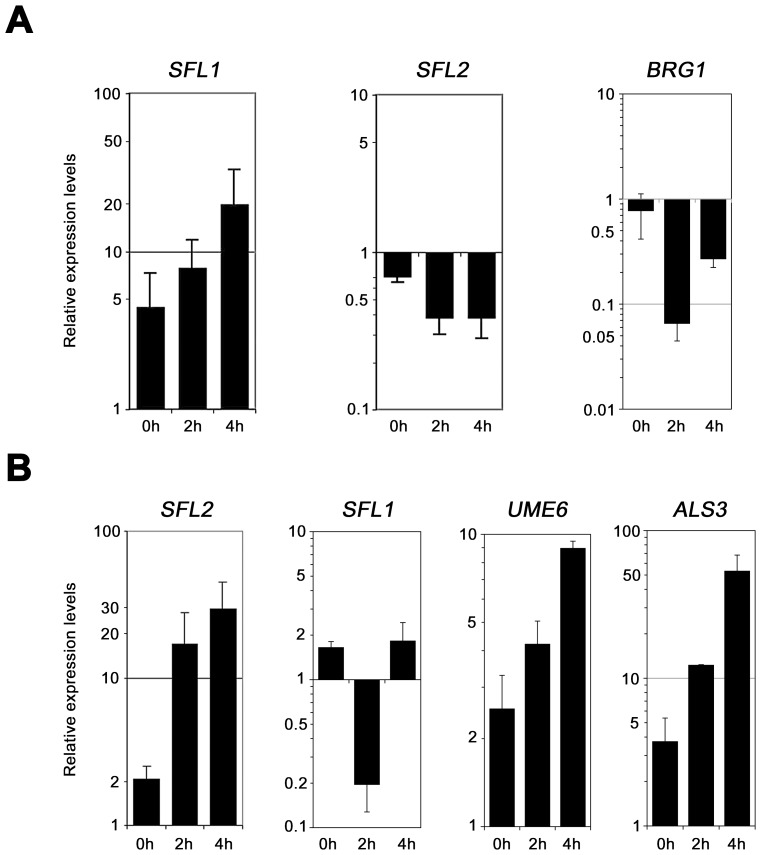
Quantitative real-time RT-PCR analysis of selected genes from *SFL1* and *SFL2* transcriptomics data. (**A**) Expression of the *SFL1*, *SFL2* and *BRG1* genes was quantified by qRT-PCR experiments in *SFL1*-deficient strains carrying or not a functional *SFL1-TAP* fusion [41] and grown during 0, 2 and 4 hours under gluconeogenic conditions (CEC2001 and CEC1997, respectively, Table 1). Expression of the *SFL2* and *BRG1* genes is repressed upon *SFL1* expression. Logarithmic scales are shown in each panel. (**B**) Expression of the *SFL2*, *SFL1*, *UME6* and *ALS3* genes was quantified by qRT-PCR experiments in an *SFL2*-deficient strain carrying or not a functional *SFL2-TAP* fusion [41] and grown during 0, 2 and 4 hours under gluconeogenic conditions (CEC1509 and CEC1535, respectively). Expression of the *SFL1* gene is repressed at time point 2 h, whereas those of *UME6* and *ALS3* are induced. Logarithmic scales are shown in each panel. Bars in each graph indicate log-transformed relative changes in RNA expression of the indicated samples as compared to the *CEF3* calibrator control (see Materials and Methods). Error bars denote standard deviations.

We independently confirmed the microarray data by qRT-PCR analyses of selected genes using homozygous *sfl1* or *sfl2* mutant strains expressing (or not) functional TAP (tandem affinity purification)-tagged *SFL1* or *SFL2* alleles [41], respectively, under the control of the *PCK1* promoter (Figure 5, Table 1). Strains were grown under gluconeogenic (P*_PCK1_*-inducing) conditions during 0, 2 and 4 hours and total RNA was isolated followed by qRT-PCR (See Materials and Methods for details). As expected, expression of *SFL1-TAP* gradually increased from time points 0 h to 4 h (Figure 5A, left panel). This increased *SFL1* expression correlated with decreased *SFL2* and *BRG1* expression (Figure 5A, middle and right panels), consistent with a negative regulation of *SFL2* and *BRG1* expression. On the other hand, P*_PCK1_*-induced *SFL2-TAP* expression (Figure 5B, left panel) correlated with decreased expression of *SFL1* (Figure 5B, *SFL1* panel) and increased expression of *UME6* and *ALS3* (Figure 5B, *UME6* and *ALS3* panels), consistent with our microarray data (Figure 4).

Taken together, our transcriptomics data reflect the antagonistic functions of Sfl1p and Sfl2p in regulating *C. albicans* morphogenesis, with *SFL1* promoting the yeast-form growth which correlates with upregulation of yeast form-specific genes and downregulation of HSGs, and *SFL2* promoting hyphal growth which correlates with upregulation of HSGs and downregulation of yeast form-specific genes.

### The Sfl1p and Sfl2p regulatory network

**Figure 6:**
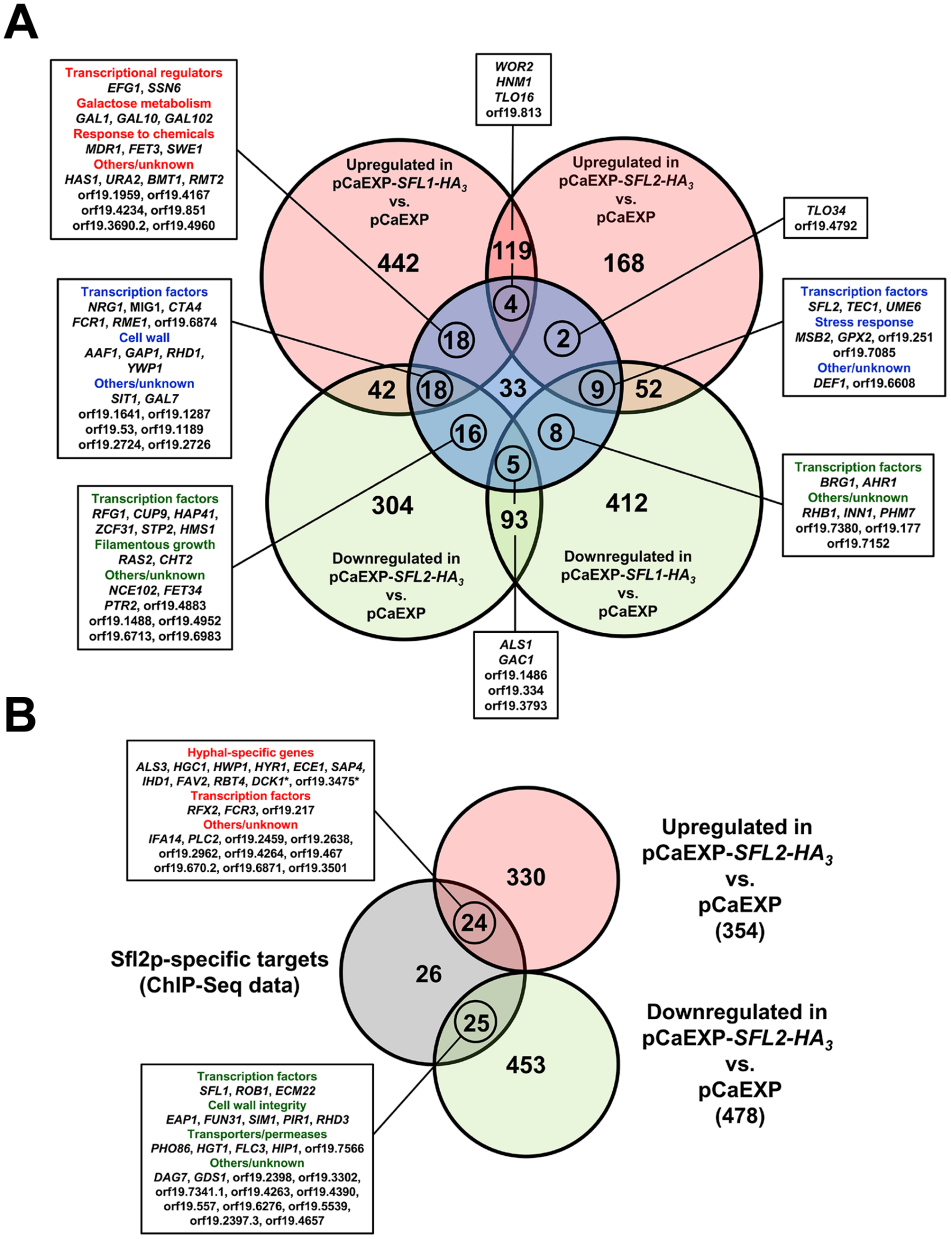
Sfl1p and Sfl2p transcriptional modules. Venn diagrams of the overlap between the genes that are modulated in (**A**) *SFL1* or *SFL2* transcriptomics (light red circles, upregulated; light green circles, downregulated; gene expression fold-change cut-off ≥1.5; P-value cut-off ≤0.05) and commonly bound by Sfl1p and Sfl2p (light blue circle) or (**B**) *SFL2* transcriptomics (light red circle, upregulated; light green circle, downregulated; gene expression fold-change cut-off ≥1.5; P-value cut-off ≤0.05) and specifically bound by Sfl2p (light grey circle). Numbers in the Venn diagrams indicate the number of genes. Circled numbers indicate the number of genes that are (**A**) both modulated in *SFL1* or *SFL2* transcriptomics data and commonly bound by Sfl1p and Sfl2p or (**B**) both modulated in *SFL2* transcriptomics data and specifically bound by Sfl2p. The name of these genes (or their orf19 nomenclature) and the functional categories to which they belong are shown in the linked boxes. *, *DCK1* is required for hyphal formation; orf19.3475 is a hyphal induced gene.

We combined the transcriptomics and the ChIP-Seq data in order to get a genome-wide view of the transcriptional modules associated with Sfl1p and Sfl2p regulatory functions (Figure 6). We were expecting to find a substantial amount of genes that are bound by Sfl1p and downregulated in pCaEXP-*SFL1-HA_3_* vs. pCaEXP microarray data, as Sfl1p is thought to act as a repressor. In line with the function of Sfl2p as an activator of hyphal growth, we were also hypothesizing that binding of Sfl2p to its targets would correlate with increased expression of these target genes. Surprisingly, among the 113 targets commonly bound by Sfl1p and Sfl2p, 40 genes were upregulated and only 22 genes were downregulated in pCaEXP-*SFL1-HA_3_* vs. pCaEXP data (Figure 6A). Conversely, 39 genes were downregulated in pCaEXP-*SFL2-HA_3_* vs. pCaEXP data and only 15 genes were upregulated (Figure 6A), indicating that Sfl1p and Sfl2p have dual transcriptional regulatory functions; acting as both transcriptional activators and transcriptional repressors.

As Sfl1p and Sfl2p respectively act as a repressor and an activator of hyphal growth, we examined the set of genes that were commonly bound by these two regulators and whose expression was both downregulated by *SFL1* and upregulated by *SFL2*. We found 9 genes matching these criteria (Figure 6A, middle right box), among which the key regulators of hyphal growth *UME6* and *TEC1*.

We also examined the set of genes that were both bound by Sfl1p and Sfl2p and upregulated in pCaEXP-*SFL1-HA_3_* vs. pCaEXP and/or downregulated in pCaEXP-*SFL2-HA_3_* vs. pCaEXP microarray data (Figure 6A, left boxes). This is consistent with Sfl1p acting as a transcriptional activator for these genes and/or Sfl2p functioning as their transcriptional repressor. Interestingly, we found that many of these genes encode (or are predicted to encode, e.g. orf19.6874) negative regulators of hyphal growth, including *SSN6*, orf19.6874 [50], *NRG1* and *RFG1* (Figure 6A, left boxes). Of particular interest, *EFG1*, the major regulator of *C. albicans* morphogenesis that functions as both a transcriptional activator and a repressor depending on the growth condition [51] was found to be upregulated by Sfl1p but not modulated in *SFL2* microarray data.

Sfl1p and Sfl2p also bound to the promoter of *BRG1*, *AHR1*, *HMS1* and *SFL2* (Figure 6A), all encoding transcriptional activators of hyphal growth. The expression of *BRG1* and *AHR1* was downregulated by Sfl1p but not modulated by Sfl2p (Figure 6A, bottom right box), whereas the expression of *HMS1* was downregulated by Sfl2p but not modulated by Sfl1p (Figure 6A, bottom left box). Interestingly, Sfl1p binding to the *SFL2* promoter correlates with decreased expression of *SFL2*, indicating a direct negative regulation of *SFL2* expression by Sfl1p (Figures 5A and 6A).

Sfl2p binding to its 75 specific target genes correlated with increased and decreased expression of 24 and 25 genes, respectively (Figure 6B). Strikingly, a significant subset of the genes that are both bound and transcriptionally induced by Sfl2p were the HSGs *ALS3*, *HGC1*, *HWP1*, *HYR1*, *ECE1*, *SAP4*, *IHD1*, *FAV2* and *RBT4* in addition to *DCK1* encoding a putative guanine nucleotide exchange factor required for filamentous growth and the hyphal induced gene orf19.3475 (Figure 6B, upper box). Moreover, Sfl2p directly upregulated genes encoding (or predicted to encode) transcription factors, including *FCR3*, encoding a positive regulator of *C. albicans* adherence [52], orf19.217, encoding a positive regulator of hyphal growth [41] and *RFX2*, encoding a regulator of DNA damage response, adhesion and virulence [53]. On the other hand, Sfl2p directly downregulated the expression of transcription factors *SFL1*, *ECM22*, *ROB1*, encoding a regulator of biofilm formation [54], and many genes involved or predicted to be involved in cell wall integrity (*EAP1*, *FUN31*, *SIM1*, *PIR1* and *RHD3*) as well as genes encoding or predicted to encode permeases or transporters (*PHO86*, putative inorganic phosphate transporter; *HGT1*, high-affinity glucose transporter; *FLC3*, putative heme transporter; *HIP1* and orf19.7566, putative amino acid transporters).

Taken together, combination of the ChIP-Seq and the transcriptomics data i) indicate that Sfl1p and Sfl2p have dual transcriptional regulatory functions, acting as both activators and repressors, ii) suggest that Sfl1p and Sfl2p antagonistic functions in regulating hyphal morphogenesis is mediated through direct transcriptional modulation of genes encoding key regulators of *C. albicans* morphogenesis, iii) show that Sfl2p additionally specifically controls the expression of HSGs and iv) reveal a direct *SFL1*-*SFL2* cross-factor negative control.

### *SFL1* and *SFL2* genetically interact with transcriptional targets encoding major regulators of morphogenesis and virulence

**Figure 7:**
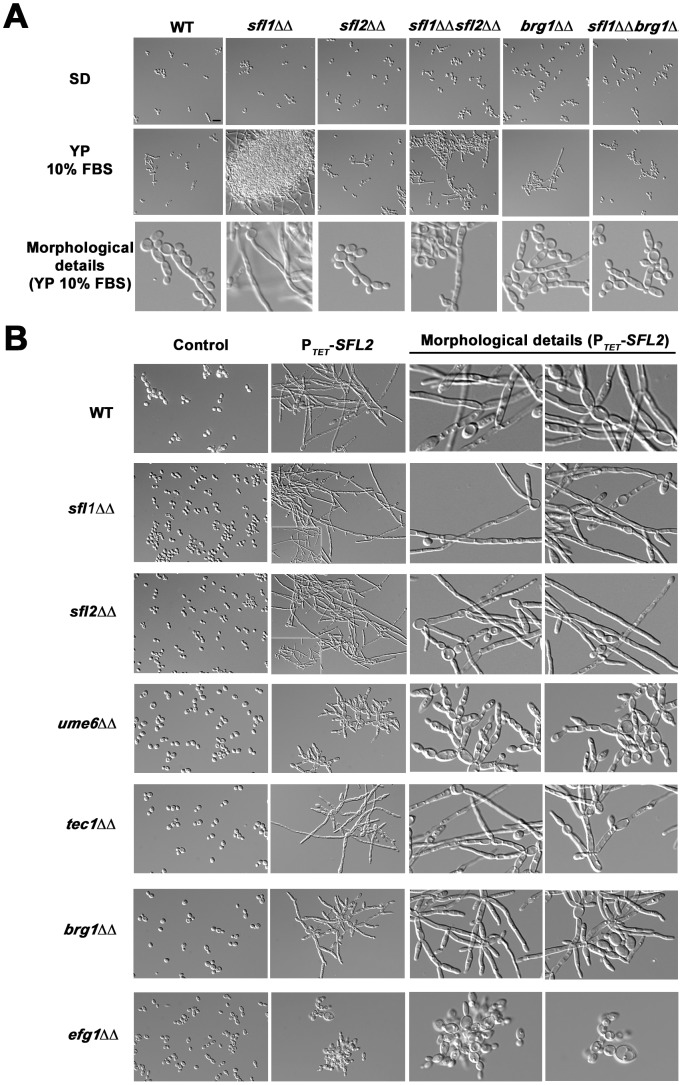
Genetic interactions of *SFL1* and *SFL2* with their transcriptional target genes encoding key regulators of hyphal development. (**A**) The wild-type SC5314 (WT) together with the homozygous *sfl1* (*sfl1*ΔΔ, CEC2001), *sfl2* (*sfl2*ΔΔ,CEC1535), *brg1* (*brg1*ΔΔ, CEC2058), the double homozygous *sfl1*, *sfl2* (*sfl1*ΔΔ *sfl2*ΔΔ, CEC2658) and *sfl1*, *brg1* (*sfl1*ΔΔ *brg1*ΔΔ, CEC2840) mutants were grown in yeast-promoting (SD at 30°C for 6 h30 min) or sub-hypha-inducing (YP 10% FBS at 30°C for 6 h30 min) conditions and observed microscopically. Scale bar = 10 µm. The detailed cell morphology of each strain grown in YP 10% FBS are shown (Morphological details, bottom panel) (**B**) The pNIMX expression system [41] was used to drive anhydrotetracycline-dependent overexpression of *SFL2* (P*_TET_*-*SFL2*) in a wild-type (WT, BWP17AH complemented for uracil auxotrophy) or in different homozygous mutant backgrounds, including *sfl1*Δ/*sfl1*Δ (*sfl1*ΔΔ), *sfl2*Δ/*sfl2*Δ (*sfl2*ΔΔ), *ume6*Δ/*ume*6Δ (*ume6*ΔΔ), *tec1*Δ/*tec1*Δ (*tec1*ΔΔ), *brg1*Δ/*brg1*Δ (*brg1*ΔΔ) and *efg1*Δ/*efg1*Δ (*efg1*ΔΔ) (Table 1). All strains were grown in YPD medium at 30°C during 18 hours in the presence of 3 µg/ml of anhydrotetracycline before microscopic examination. As a control, the same growth conditions were also used with all strain backgrounds carrying the empty plasmid (CIp10, Control). Two different fields with detailed cell morphology of each strain overexpressing *SFL2* are shown (Morphological details, right panels).

Our finding that Sfl1p and Sfl2p directly control the expression of master regulators of *C. albicans* morphogenesis and virulence fostered us to assess the genetic interactions between *SFL1*, *SFL2* and these target genes. Data mining of our ChIP-Seq and transcriptomics results showed that Sfl1p directly negatively regulates *SFL2* expression (Figures 3, 5A and 6A). Moreover, Sfl1p directly negatively regulates the expression of *BRG1* (Figures 3, 5A and 6A), encoding a major regulator of hyphal growth. This suggests that *SFL1* represses filamentation through, at least, direct transcriptional repression of the *SFL2* and *BRG1* genes. To test this hypothesis, we constructed *sfl1*Δ/*sfl1*Δ, *sfl2*Δ/*sfl2*Δ and *sfl1*Δ/*sfl1*Δ, *brg1*Δ/*brg1*Δ double mutants and tested their ability to form hyphae (Figure 7A). All strains displayed yeast-form growth in SD medium at 30°C (Figure 7A, upper panels). In YP 10% FBS medium at 30°C (Figure 7A, middle and lower panels), which induces moderate filamentation, the homozygous *sfl1* mutant displayed highly dense cell aggregates of a mixture of hyphae and long pseudohyphae (Figure 7A, middle and lower panels), consistent with the function of *SFL1* as a transcriptional repressor of filamentous growth. Interestingly, deletion of *SFL2* or *BRG1* in the *sfl1* mutant strongly reduced filamentous growth as well as cell aggregation (Figure 7A, middle and lower panels), with the *sfl1 sfl2* double mutant cells growing as both yeast form and long to medium-size pseudohyphae and the *sfl1 brg1* double mutants growing as both yeast form and short pseudohyphae (Figure 7A, middle and lower panels). Single homozygous *sfl2* and *brg1* mutants showed phenotypes that were similar to those of the parental wild-type cells (Figure 7A, middle and lower panels).

We showed that Sfl2p directly upregulated *UME6* and *TEC1* expression (Figures 3, 5B and 6A), while specifically directly downregulating the expression of *SFL1* (Figures 3, 5B and 6B), suggesting that *SFL2* controls hyphal induction through at least *UME6*, *TEC1* and *SFL1*. We tested the effect of overexpressing *SFL2* on *C. albicans* morphogenesis in strains carrying the single homozygous deletions *sfl1*, *sfl2*, *ume6*, *tec1*, *brg1* and *efg1* (Figure 7B). We and others previously showed that *SFL2* overexpression in non-hypha-inducing conditions promotes hyphal growth [39], [40]. We used the pNIMX system [41] to drive high levels of *SFL2* expression in the above-mentioned strain backgrounds grown in rich medium (Figure 7B). Overexpression of *SFL2* in the wild-type strain strongly induced filamentation, with cells displaying long pseudohyphae (Figure 7B, top panels). Interestingly, *SFL2*-driven filamentation was increased in the *sfl1*Δ/*sfl1*Δ mutant, as compared to that in the wild-type or the *sfl2*Δ/*sfl2*Δ strains (Figure 7B, compare the zoomed-out regions in lower left corners). Most of the *sfl1* mutant cells overexpressing *SFL2* formed longer hyphae and pseudohyphae than those observed in the equivalent *sfl2* mutants (Figure 7B), suggesting that Sfl2p induces filamentous growth in part through repression of *SFL1* expression. Conversely, filamentation was strongly reduced in the *ume6*Δ/*ume6*Δ strain, moderately reduced in either the *tec1*Δ/*tec1*Δ or *brg1*Δ/*brg1*Δ mutants and abolished in the *efg1*Δ/*efg1*Δ strain (Figure 7B). The *ume6* mutants overexpressing *SFL2* formed significantly shorter pseudohyphae than those of the equivalent *tec1* and *brg1* mutants (Figure 7B).

Taken together, our results suggest that Sfl1p represses filamentation through at least direct negative regulation of *SFL2* and *BRG1* expression and indicate that Sfl2p regulates hyphal growth partly through *UME6*, *TEC1* and *BRG1* and totally through *EFG1*.

### Motif discovery analyses suggest functional interactions between Sfl1p, Sfl2p, Efg1p and Ndt80p

Many observations support the hypothesis that Sfl1p and Sfl2p recognize different binding motifs. First, although sharing common transcriptional targets, Sfl1p and Sfl2p peak signals are distributed differently along many of their common target promoters (Figure 2B, middle panel as an example). Second, Sfl2p binds specifically to the promoter of 75 targets (Figure 2B, bottom panel as an example). Third, recent data by Song *et al.* suggested that Sfl1p and Sfl2p mediate their functional divergence through their HSF-type DNA binding domain [39], suggesting divergent binding sites.

**Figure 8:**
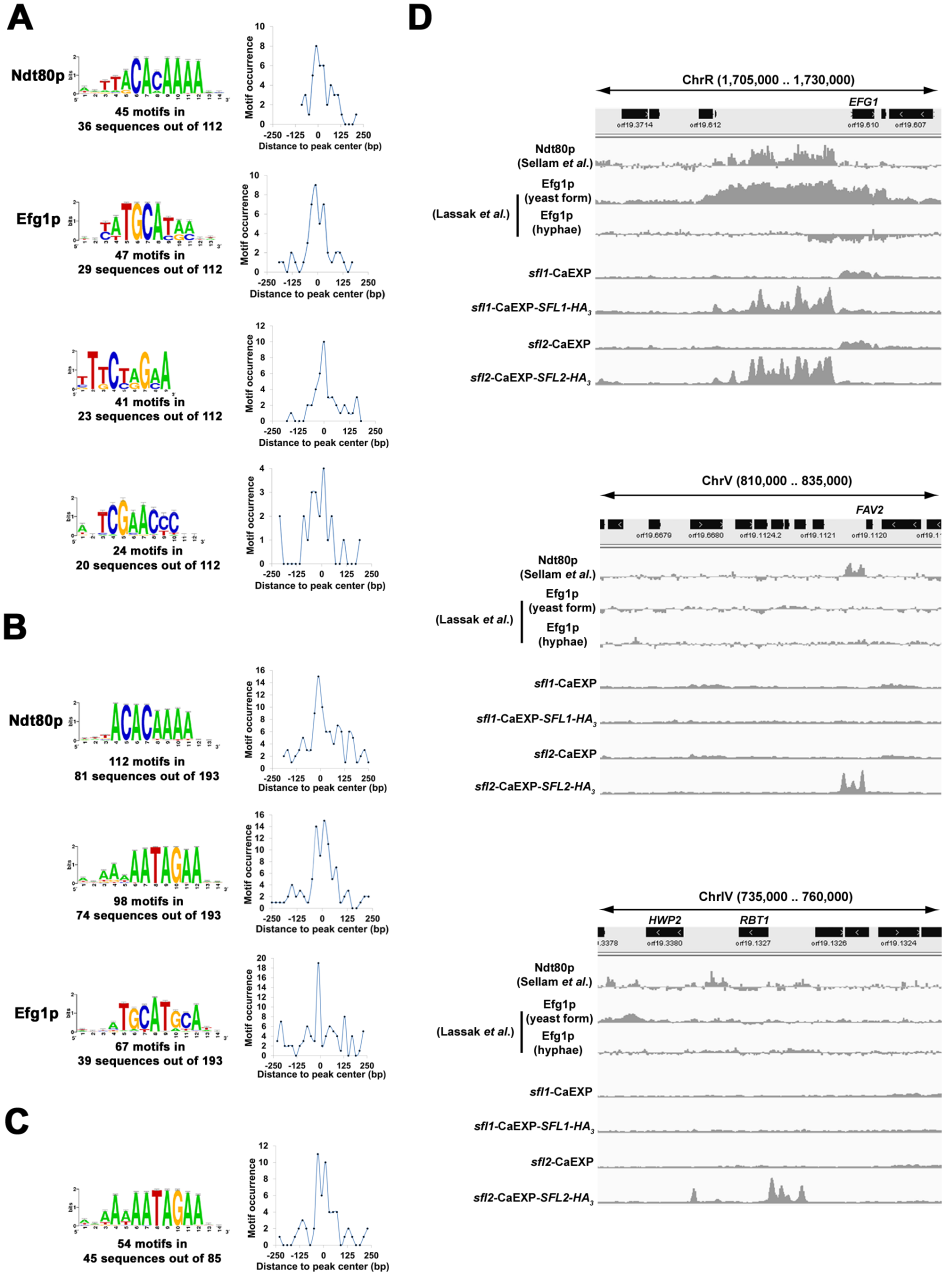
Sfl1p and Sfl2p binding locations overlap with those of Ndt80p and Efg1p. (**A, B and C**) Motif discovery analyses of Sfl1p and Sfl2p binding data. Motif logos of conserved sequences in (**A**) Sfl1p- and (**B**) Sfl2p-enriched DNA fragments as well as in (**C**) fragments overlapping with binding regions that are specific to Sfl2p. DNA sequences encompassing ±250 bp around peak summits in Sfl1p or Sfl2p binding data were used as input for motif discovery using two independent motif discovery algorithms, the RSA-tools (RSAT) peak-motifs (http://rsat.ulb.ac.be/rsat/, [55]) and SCOPE (genie.dartmouth.edu/scope/, [56]) (See Materials and Methods for details). High scoring motifs from either SCOPE or RSAT algorithms are shown. These include the Ndt80p and Efg1p binding motifs, suggesting a functional interaction between Sfl1p, Sfl2p, Ndt80p and Efg1p. The distribution of motif occurrences in the input sequences are shown at the right of each motif panel. Plotted are the number of occurrences of each motif (*y*-axis, motif occurrence) at a given position relative to peak center (distance to peak center in base pairs, *x*-axis). (**D**) Overlap of Ndt80p and Efg1p binding with Sfl1p and Sfl1p occupancies at selected locations from the *C. albicans* genome (selected genome interval shown above each panel). Genome-wide location data from Sellam *et al.* (Ndt80p, from 59-bp tiling array data, one of the two replicates of the study is shown [57]) and Lassak *et al.* (Efg1p, from 50–75-mer tiling array data for Efg1p binding in cells grown under yeast form and during hyphal induction [51], one of the three replicates in each condition is shown) are used to compare Ndt80p and Efg1p binding profiles to those of Sfl1p and Sfl2p (read counts in 10 bp windows from wiggle files of Sfl1p and Sfl2p binding data were used).

We performed motif-enrichment analyses using DNA sequences encompassing ±250 bp around peak summits in Sfl1p (Figure 8A) and Sfl2p (Figure 8B) binding data. Two independent motif discovery algorithms, the RSA-tools (RSAT) peak-motifs (http://rsat.ulb.ac.be/rsat/, [55]) and SCOPE (genie.dartmouth.edu/scope/, [56]) were used (See Materials and Methods for details). Strikingly, the highest scoring motifs in Sfl1p-enriched sequences included the Ndt80p (5′-ttACACAAA-3′, mid-sporulation element, lowercase letters represent nucleotides with low-frequency occurrence) and the Efg1p (5′-taTGCAta-3′) binding motifs [51], [54], [57] in addition to two high scoring motifs, 5′-TtCtaGaA-3′ and 5′-TCGAACCC-3′, carrying GAA triplets that are characteristic of HSEs (Figure 8A, shown are motifs found using the global overrepresentation of words relative to control sequences, significance index score (i.e. −log_10_ *E*-value) >10 for RSAT analyses and >25 for SCOPE analyses). Ndt80p is a transcription factor that controls the expression of genes involved in many cellular processes, including drug resistance, cell separation, morphogenesis and virulence through the recognition of mid-sporulation elements on the promoter of its targets [57], [58]. This suggests the presence of functional interactions between Sfl1p, Efg1p and Ndt80p and proposes that Sfl1p binds to two different motifs or that an additional factor binds either 5′-TCGAACCC-3′ or 5′-TtCtaGaA-3′. We searched the YeTFaSCo and the JASPAR databases for similarity with known transcription factor binding sites [59], [60]. Interestingly, the 5′-TtCtaGaA-3′ sequence was strongly similar to the *S. cerevisiae* Hsf1p motif (*P* = 3.856×10^−04^, using YeTFaSco), while database searches did not identify any known motif that closely resembled the 5′-TCGAACCC-3′ sequence (data not shown). On the other hand, we found 3 high-scoring motifs in Sfl2p-enriched sequences, including the Efg1p and Ndt80p binding motifs as well as the GAA-containing sequence, 5′-aaNAATAGAA-3′ (where N represents any nucleotide; shown are motifs found using the position-analysis program, significance index score >5) (Figure 8B). To confirm that the 5′-aaNAATAGAA-3′ motif was specific to Sfl2p, we performed motif discovery analyses using DNA sequences encompassing ±250 bp around peak summits of the regions specifically bound by Sfl2p and found the similar high-scoring motif 5′-aANAATAGAA-3′ (Figure 8C). The 5′-aANAATAGAA-3′ motif shows moderate similarity with the *S. cerevisiae* Sfl1p and Mga1p motifs (scores = 17.75 and 17.36, respectively using the JASPAR database). All these identified motifs were distributed preferentially around the center of the sequences corresponding to peak locations (Figures 8A, 8B and 8C), suggesting that Sfl1p, Sfl2p, Efg1p and Ndt80p binding sites were very close to each other.

To determine if Efg1p and Ndt80p binding sites overlapped with the genome-wide occupancies of Sfl1p and Sfl2p, we compared Efg1p and Ndt80p binding profiles [51], [57] to those of Sfl1p and Sfl2p (Figure 8D). Ndt80p binding was resolved by Sellam *et al.* under yeast-form growth conditions at 30°C [57], whereas Efg1p binding was analysed by Lassak *et al.* during both yeast-form growth (30°C) and hyphal induction (YP serum at 37°C) [51]. Strikingly, a high proportion of Sfl1p and Sfl2p binding sites overlapped with those of Ndt80p (Figure 8D), whereas Efg1p binding overlap was less frequent and depended on the morphological state of *C. albicans*, with rare or no overlap under hyphal induction and increased overlap under yeast-form growth (Figure 8D). Roughly, 90% of Sfl1p and Sfl2p common targets were bound by both Ndt80p and Efg1p (Figure 8D, upper panel as an example), whereas ∼10% (10 out of 113 common targets) were bound by Ndt80p but not Efg1p. In at least two cases, Sfl1p and Sfl2p occupancy to common targets overlapped only with Efg1p binding: the promoter regions of *SIS1* and *PDE1*. On the other hand, ∼47% of Sfl2p specific targets were bound by both Ndt80p and Efg1p, whereas ∼42% overlapped only with Ndt80p binding (Figure 8D, middle panel as an example). On rare occasions (∼11%), Sfl2p did not show significant overlap with the binding of any of the three regulators (Figure 8D, bottom panel as an example).

Taken together, our results indicate that Sfl1p and Sfl2p bind to DNA via divergent motifs and suggest the co-binding of transcription factors Efg1p and Ndt80p to many Sfl1p and Sfl2p target promoters, either concomitantly or successively, depending on growth conditions.

### The Efg1p protein binds to the promoter of many Sfl1p and Sfl2p targets and co-immunoprecipitates with Sfl1p and Sfl2p, *in vivo*

**Figure 9:**
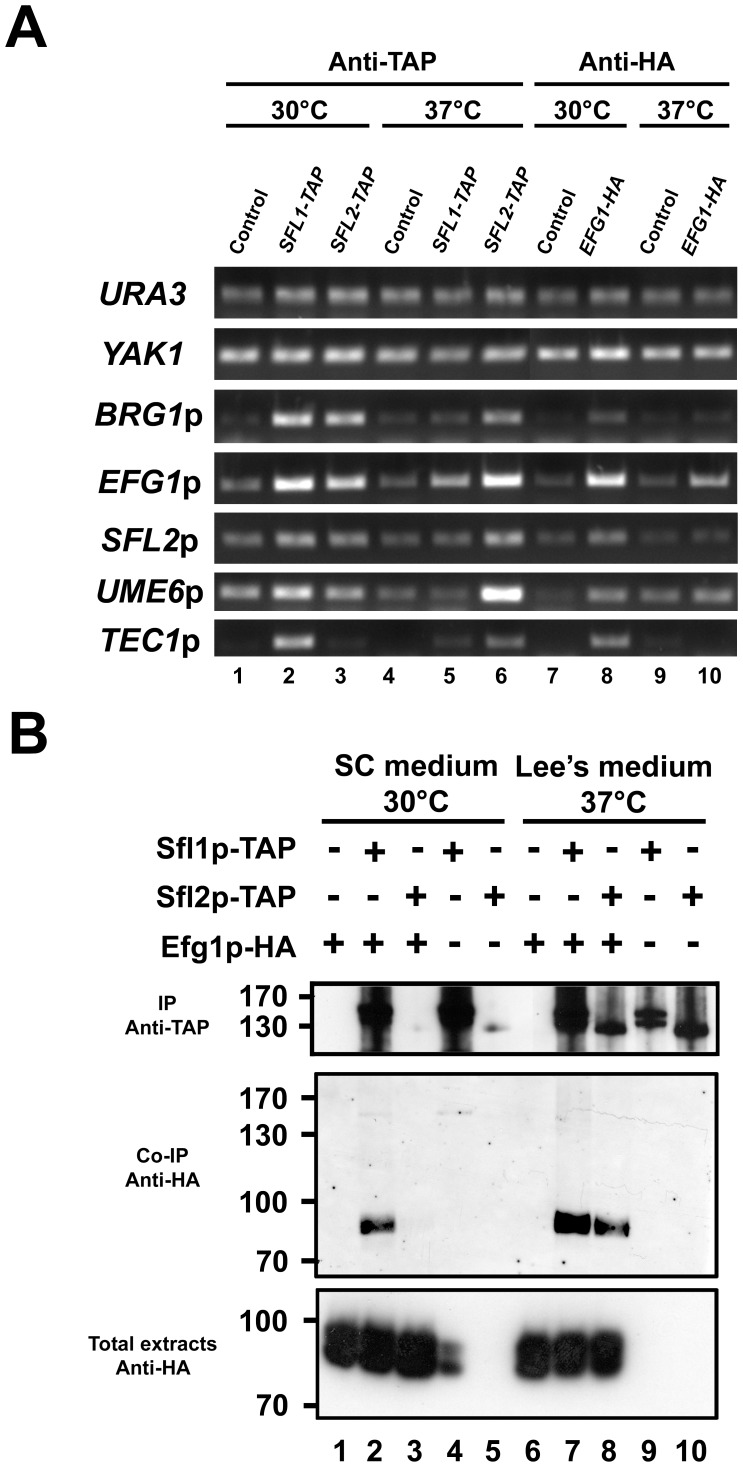
Efg1p binds to the promoter of many Sfl1p and Sfl2p targets and co-immunoprecipitates with Sfl1p and Sfl2p, *in vivo*. (**A**) ChIP-PCR assay of selected Sfl1p and Sfl2p target promoters. Strains SFL1-TAP (CEC1922), SFL2-TAP (CEC1918) and EFG1-HA (HLCEEFG1) were grown in SC medium at 30°C (30°C) or in Lee's medium at 37°C (37°C) together with the SC5314 control strain (Control) during 4 h before being subjected to chromatin immunoprecipitation (Anti-TAP, Anti-HA) followed by PCR using primers specific to the indicated promoter regions. The *URA3* and *YAK1* genes were used as negative controls for ChIP enrichment. (**B**) Co-Immunoprecipitation of Efg1p with Sfl1p and Sfl2p. Strains coexpressing *SFL1-TAP* and *EFG1-HA* (Lanes 2 and 3) or *SFL2-TAP* and *EFG1-HA* (Lanes 7 and 8) or controls (Lanes 1 and 6, *EFG1-HA* only; lanes 4 and 9, *SFL1-TAP* only; lanes 5 and 10, *SFL2-TAP* only) were cultivated in SC medium at 30°C or in Lee's medium at 37°C before crosslinking with formaldehyde. Total extracts were incubated with Dynal PanMouse IgG beads directed against TAP epitope tag prior to washing and Western blotting using anti-TAP (IP Anti-TAP, 10% of the beads/total extracts mixture) and anti-HA (Co-IP Anti-HA) antibodies. A portion of the total cell extracts (∼2%) was included to verify the presence of the Efg1p-HA fusion (Total extracts Anti-HA).

Our bioinformatic analyses suggested the co-binding of Efg1p to many Sfl1p and Sfl2p target promoters. To test whether Sfl1p, Sfl2p and Efg1p concomitantly bind to common targets *in vivo*, strains individually expressing chromosomally TAP-tagged Sfl1p and Sfl2p (strains SFL1-TAP and SFL2-TAP, Table 1) and HA-tagged Efg1p (strain HLCEEFG1, [18], Table 1) under the control of their endogenous promoter were grown in SC medium at 30°C (yeast form-promoting condition) or in Lee's medium at 37°C (filamentous form-promoting condition) during 4 h before being subjected to ChIP-PCR analyses to detect differential binding of the three transcription factors to the promoter of selected Sfl1p and Sfl2p targets (*BRG1*, *EFG1*, *SFL2*, *UME6* and *TEC1*, Figure 9A, see Materials and Methods for details). All strains displayed similar hyphal growth phenotypes at 37°C in Lee's medium, whereas the yeast form growth phenotypes were similar for cells grown in SC medium at 30°C (Figure S1A). Immunoblotting confirmed the expression of the different fusion proteins under the corresponding growth conditions (Figure S1B). As expected, Sfl1p and Efg1p binding was detected at all tested promoters in SC medium at 30°C (Figure 9A, compare lanes 1 and 7 to lanes 2 and 8, respectively). Conversely, in Lee's medium at 37°C, Sfl1p and Efg1p binding was less efficient (Figure 9A, Sfl1p binding, compare lanes 1 and 2 to lanes 4 and 5; Efg1p binding, compare lanes 7 and 8 to lanes 9 and 10). Similarly, Sfl2p binding was detected at all tested promoters in Lee's medium at 37°C (Figure 9A, compare lane 4 to lane 6), whereas in SC medium at 30°C, Sfl2p binding was less efficient (Figure 9A, compare lanes 4 and 6 to lanes 1 and 3).

To further explore the functional interaction between Sfl1p, Sfl2p and Efg1p, we sought to verify if the Efg1p protein could be co-immunoprecipitated with Sfl1p or Sfl2p *in vivo*. To this end, we generated strains co-expressing C-terminally TAP-tagged Sfl1p or Sfl2p and HA-tagged Efg1p (AVL12-SFL1-TAP and AVL12-SFL2-TAP, respectively, Table 1) under the control of their chromosomal promoter together with control strains carrying individual Sfl1p-TAP, Sfl2p-TAP or Efg1p-HA fusions (strains SFL1-TAP, SFL2-TAP and AVL12-pHIS, Table 1, see Materials and Methods). Strains were grown during 4 h in SC medium at 30°C or in Lee's medium at 37°C, followed by crosslinking with formaldehyde to stabilize protein complexes and total extracts were incubated with IgG-coated beads for immunoprecipitation of the Sfl1p-TAP or Sfl2p-TAP proteins in the corresponding strain backgrounds. Immunoblotting with an anti-TAP antibody (Figure 9B, IP, Anti-TAP panel) allowed to detect the Sfl1p-TAP signal in beads incubated with extracts from strains carrying the *SFL1-TAP* allele irrespective of the growth conditions (i.e. in both SC medium at 30°C and Lee's medium at 37°C) (Figure 9B, IP, Anti-TAP panel, lanes 2, 4, 7 and 9). On the other hand, very low amounts of the Sfl2p-TAP protein fusion were detected in beads incubated with extracts from strains carrying the *SFL2-TAP* allele and grown in SC medium at 30°C (Figure 9B, IP Anti-TAP panel, lanes 3 and 5), however, the Sfl2p-TAP signal strongly increased in Lee's medium at 37°C (Figure 9B, Anti-TAP panel, compare lanes 3 and 5 to lanes 8 and 10). Interestingly, immunoblotting of the bound fractions with an anti-HA antibody (Co-IP, Anti-HA panel) allowed to detect Efg1p-HA co-immunoprecipitation with Sfl1p-TAP under both growth conditions: in SC medium at 30°C and in Lee's medium at 37°C (Figure 9B, CoIP, Anti-HA panel, lanes 2 and 7). Efg1p-HA co-immunoprecipitation with Sfl2p-TAP was barely detectable in SC medium at 30°C but was significantly enhanced in Lee's medium at 37°C, a condition that triggers increased expression of Sfl2p (Figure 9B, CoIP, Anti-HA panel, compare lane 3 to lane 8). As expected, Efg1p-HA was undetectable from beads incubated with strains individually expressing *EFG1-HA*, *SFL1-TAP* or *SFL2-TAP* (Figure 9B, lanes 1, 4, 5, 6, 9 and 10).

Taken together, our results show that i) the Efg1p protein binds to many Sfl1p and Sfl2p targets, *in vivo* and ii) Both Sfl1p and Sfl2p proteins physically associate with Efg1p, *in vivo*.

## Discussion

The ChIP-Seq and transcriptomics technologies are powerful *in vivo* approaches that, when combined, allow to provide mechanistic insights into the function of transcriptional regulators. When associated with both genetic and physical interaction analyses, the overall generated data are cross-validated and provide a comprehensive view of the regulatory interactions within transcriptional networks. They also shed more light into the epistatic relationships to explain the phenotypes associated with transcription factor function. In the present report, we used such approaches to decipher the regulatory network of two HSF-type transcription factors, Sfl1p and Sfl2p, both required for *C. albicans* virulence but with antagonistic functions in regulating *C. albicans* morphogenesis. One limitation of our ChIP-Seq design was the use of ectopic promoter-driven expression of the *SFL1-HA_3_* and *SFL2-HA_3_* alleles (Figure 1). This may drive non physiological expression levels and some of the transcriptional changes and promoter occupancies may be altered from the situation where the genes are expressed from their endogenous promoters. Nevertheless, phenotypic analyses suggested that at least P*_MET3_*-driven expression of *SFL2-HA_3_* imparts filamentous growth in a manner similar to the wild-type SC5314 strain (Figure 1C). Furthermore, we generated strains expressing TAP-tagged *SFL1* and *SFL2* from their endogenous promoter and ChIP experiments using these strains confirmed some of our data that used the P*_MET3_* expression system (Figure 9A).

**Figure 10:**
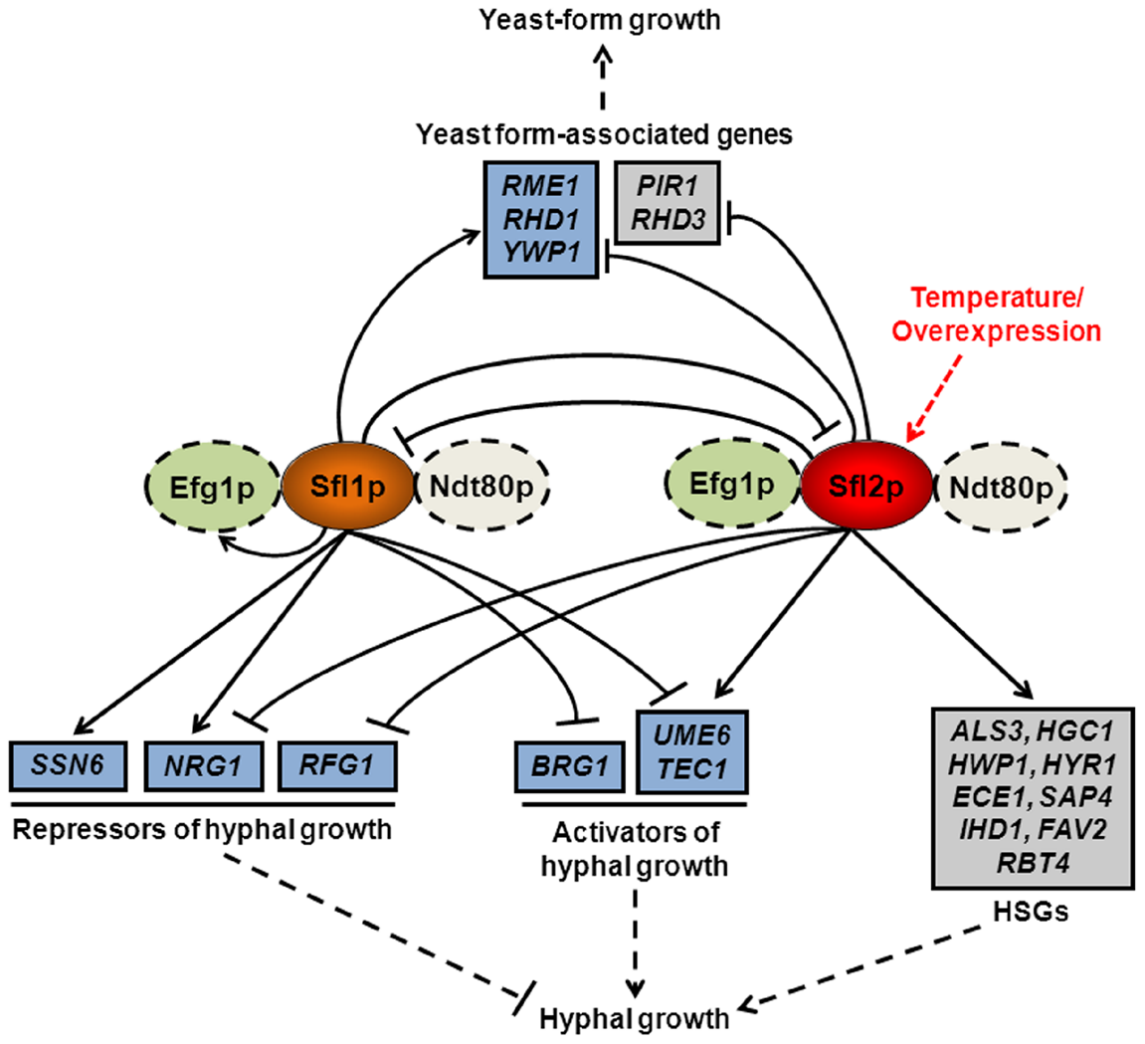
Model of Sfl1p and Sfl2p regulatory network. Sfl2p (red oval), which induces hyphal growth in response to temperature increase or upon overexpression (red dashed arrow), and Sfl1p (orange oval) bind directly, together with Efg1p and Ndt80p depending on growth conditions (green and white ovals, respectively; dashed lines indicate hypothetical physical and/or functional interaction), to the promoter of common (blue boxes) target genes encoding major transcriptional activators (*UME6*, *TEC1* and *BRG1*) or repressors (*NRG1*, *RFG1*, *SSN6*) of hyphal growth as well as to the promoter of genes associated with yeast-form growth (*RME1*, *RHD1* and *YWP1*) and modulate the expression of many of them (for simplicity, only modulatory direct interactions are shown i.e. both binding at and transcriptional modulation of a given target; arrowed lines indicate direct upregulation whereas blunt lines indicate direct downregulation). On the other hand, Sfl2p directly upregulates the expression of specific targets (grey boxes), including a high proportion of hyphal-specific genes (HSGs), while exerting a direct negative regulation on the expression of yeast-form associated genes (*PIR1* and *RHD3*). Sfl1p and Sfl2p also exert a direct negative regulation on the expression of each other. The execution of Sfl1p or Sfl2p transcriptional control inputs allows to regulate the commitment (dashed line; blunt, inhibition; arrowed, activation) of *C. albicans* to form hyphae or yeast-form cells.

Our data allow to propose a model of Sfl1p and Sfl2p transcriptional network (Figure 10, for simplicity only binding associated with transcriptional modulation is shown) as well as a mechanism whereby Sfl1p and Sfl2p antagonistically regulate the yeast-to-hyphae transition (see below). Sfl2p, which responds to temperature increase, and Sfl1p bind to the promoter of common target genes (blue boxes in Figure 10) belonging to at least 3 functional groups involved in morphogenesis: transcriptional repressors of hyphal growth (*SSN6*, *NRG1*, *RFG1*, others), transcriptional activators of hyphal growth (*BRG1*, *UME6*, *TEC1*, others) and yeast-form associated genes (*RME1*, *RHD1*, *YWP1*, others). While Sfl1p exerts direct negative and positive regulation on the expression of activators (*BRG1*, *UME6*, *TEC1*) and repressors (*SSN6*, *NRG1*) of hyphal growth, respectively, Sfl2p directly upregulates and downregulates the expression of positive (*UME6*, *TEC1*) and negative (*RFG1*, *NRG1*) regulators of hyphal growth, respectively (Figure 10). Additionally, Sfl1p directly upregulates the expression of yeast-form associated genes (*RME1*, *RHD1* and *YWP1*) whereas Sfl2p directly downregulates their expression (Figure 10). Moreover, Sfl1p and Sfl2p directly negatively regulate the expression of each other (Figure 10). As stated above, this model is consistent with the genetic interaction analyses performed between *SFL1* (genetically interacts with at least *BRG1* and *SFL2*), *SFL2* (genetically interacts with at least *UME6*, *TEC1* and *BRG1*) and their target genes (Figure 7). Importantly, on the other hand Sfl2p exclusively binds to the promoter of specific target genes that belong to at least 2 functional groups involved in morphogenesis: HSGs (*ALS3*, *HGC1*, *HWP1*, *HYR1*, *ECE1*, *SAP4*, *IHD1*, *FAV2*, *RBT4*) and yeast-form specific genes (*PIR1*, *RHD3*) (Figure 10). We propose that binding of Sfl1p and Sfl2p to a high proportion of their transcriptional targets occurs with additional binding of transcription factors Ndt80p and/or Efg1p, depending on growth conditions (Figures 8, 9 and 10), presumably through direct or indirect physical interaction (Figures 8 and 9, see below). One could speculate that the requirement of a functional *EFG1* gene for Sfl1p and Sfl2p abilities to regulate morphogenesis under specific growth conditions (Figure 7 and [39]) could be explained by the need for Efg1p co-binding and/or physical interaction, as suggested by our study (Figures 7, 8 and 9). Indeed, we show here that Efg1p co-immunoprecipitates, *in vivo*, with Sfl1p and Sfl2p and binds to the promoter of many Sfl1p and Sfl2p target genes (Figure 9). On the other hand, our finding that Sfl2p binds exclusively to specific targets, including a high proportion of HSGs (Figure 6), provides additional insight into *SFL2* function. This might explain, for instance, why *SFL2* was able to bypass the need of *EFG1* and *FLO8* to induce hyphal growth in embedded conditions at 37°C [39]. We are currently testing whether Sfl1p and Sfl2p binding to their targets requires the presence of functional *EFG1* or *NDT80* genes. Overall, we propose that the execution of these single (including *SFL1*-*SFL2* cross-factor negative control) and multiple input motifs in Sfl1p or Sfl2p transcriptional network dictates the commitment of the *C. albicans* cells to form hyphae or yeast-form cells. This model is consistent with Sfl1p and Sfl2p acting as “switch on/off” proteins, with Sfl1p directly turning off the expression of positive regulators of hyphal growth while turning on the expression of both yeast-form associated genes and genes encoding repressors of hyphal development, whereas Sfl2p directly turns on the expression of HSGs and positive regulators of hyphal growth while turning off the expression of yeast-form associated genes as well as negative regulators of hyphal development (Figure 10).

The mechanisms whereby HSF-type transcription factors activate transcription involve homotrimerization, post-translational modifications (e.g. phosphorylation, others) as well as interaction with multiple protein partners, followed by recruitment of the co-activating mediator complex and initiation of the transcriptional process [61]. This mechanism may include or not nuclear translocation, as many HSFs were shown to reside in the nucleus under both activating and non-activating conditions or to be imported to the nucleus following activation [61]. It was shown that Sfl1p is constitutively localized to the nucleus under both yeast- and hyphae-promoting conditions and irrespective of temperature levels [37], [38], whereas an Sfl2p-GFP fusion was undetectable at 25°C but displayed nuclear localization at 37°C [39]. Moreover, *SFL2* RNA levels were undetectable by Northern blotting at either 25°C or 30°C, but were greatly enhanced upon temperature increase [39] and this correlated with Sfl2p protein level variations [39]. Indeed, we show here that in SC medium at 30°C, Sfl2p protein levels are low, but are significantly enhanced upon temperature increase to 37°C in Lee's medium (Figure S1B). Moreover, we show that Sfl2p binding is more stable at 37°C in Lee's medium as compared to 30°C in SC medium, and *vice versa* for Sfl1p (Figure 9A). Based on these observations, we propose the following model of Sfl1p/Sfl2p activation: Sfl1p binds to its transcriptional targets to maintain the yeast form growth at low temperature by directly modulating the expression of genes involved in morphogenesis (Figure 10). A temperature increase to 37°C leads to an increase in both Sfl2p expression and binding to the promoter of Sfl1p targets in addition to specific targets (including HSGs) and induction of the hyphal development program (Figure 10). As we show here that Sfl1p and Sfl2p act as both activators and repressors of gene expression (Figures 6 and 10), it is likely that they alternatively recruit (directly or indirectly) co-repressors (e.g. Tup1p-Ssn6p) and co-activators (e.g. mediator-Swi/Snf complex) at different binding sites to regulate morphogenesis. Our observation that Sfl2p binds to its own promoter, but not Sfl1p (Figures 3, 6Aand 10) is consistent with this model as *SFL2* may undergo auto-induction which would lead to a rapid, amplified and sustained expression of *SFL2*, allowing an efficient response to temperature increase. On the other hand, *SFL1* expression, protein levels and nuclear localization remain constant under various conditions [38], which may dispense the need for autoregulation. The *SFL1*-*SFL2* cross-factor negative control is also consistent with this model. Under low temperature conditions, Sfl1p directly turns off *SFL2* expression to prevent activation of hyphal growth. Upon a temperature increase, *SFL2* expression is enhanced and Sfl2p binds to the *SFL1* promoter to turn off *SFL1* expression. This allows to relieve Sfl1p-mediated repression, thus contributing to activation of the hyphal development program.

Our motif discovery analyses suggested that Ndt80p co-binds together with Efg1p to the promoter of Sfl1p and Sfl2p targets (Figure 8). We also strikingly found that a high proportion of Sfl1p and Sfl2p binding sites overlapped with those of Ndt80p and/or Efg1p (Figure 8). However, since the Ndt80p ChIP-on-chip was performed on yeast-form grown cells at 30°C [57], one cannot exclude the possibility that Ndt80p binding is altered/lost upon hyphal induction, as is obviously the case for Efg1p ([51] and Figures 8D and 9A). Ndt80p occupies the promoter region of roughly a quarter of total *C. albicans* genes under yeast-form growth conditions, suggesting wide functions for Ndt80p [57]. Indeed, it was shown that Ndt80p regulates different processes including drug resistance, cell separation, hyphal differentiation, biofilm formation and virulence [54], [57], [58]. Importantly, the *C. albicans ndt80*Δ/*ndt80*Δ mutant is unable to form true hyphae under different filamentation-inducing conditions and, in the presence of serum at 37°C, it fails to activate the expression of HSGs, including *HWP1*, *ECE1*, *RBT4*, *ALS3*, *HYR1* and *SAP4* [58], all directly regulated by Sfl2p (Figure 6), as well as the transcription factor-encoding genes *TEC1* and *UME6* which are both directly modulated by Sfl1p and Sfl2p (Figure 6). Additionally, under the same growth conditions, the homozygous *ndt80* mutant was unable to downregulate the yeast form-associated genes *YWP1*, *RHD3*, *RHD1* and the transcriptional repressor-encoding gene *NRG1* [58], which are also direct targets of Sfl1p or Sfl2p (Figure 6). These observations, together with our findings that i) Ndt80p binding motif was enriched among Sfl1p and Sfl2p bound sequences and that ii) a significant proportion of its genome-wide binding profile overlapped with Sfl1p and Sfl2p binding, suggest that Sfl1p, Sfl2p and Ndt80p cooperatively regulate *C. albicans* morphogenesis in response to temperature variation. Whether Sfl1p and Sfl2p regulate this process through physical interaction with Ndt80p and the associated sequence of molecular events occurring during the yeast-to-hyphal switch await further characterization. On the other hand, we found that Efg1p binding also overlapped with that of Sfl1p and Sfl2p, at a lesser extent, though, as compared to Ndt80p binding (Figure 8). It is intriguing that Efg1p binding undergoes alteration following the induction of hyphal development ([51] and Figures 8D and 9A). Our examination of Efg1p binding data by Lassak *et al.* [51] together with our ChIP experiments (Figure 9A) suggest that Efg1p binding to many targets is decreased/altered upon hyphal induction. We show here that during yeast-form growth, at low temperature, Efg1p co-immunoprecipitates with Sfl1p but not with Sfl2p, presumably due to the low levels of Sfl2p at low temperature (Figure 9B). One could speculate that, at low temperature, Sfl1p associates directly or indirectly with Efg1p on the promoter of its targets to repress hyphal development. Following a temperature increase, both Sfl2p levels and Sfl2p DNA binding are enhanced (Figures S1 and 9A), which in turn activates the hyphal development program. Although Efg1p binding is altered upon hyphal induction, Efg1p co-immunoprecipitated with Sfl2p (Figure 9B) at 37°C in Lee's medium, which may explain Sfl2p dependency on *EFG1* to regulate morphogenesis under certain conditions. Nobile *et al.* elegantly showed that an intricate transcriptional network involving Ndt80p, Efg1p, Brg1p, Bcr1p, Rob1p and Tec1p controls biofilm development in *C. albicans* [54]. Interestingly, with the exception of *BCR1*, all genes encoding these regulators are direct targets of Sfl1p or Sfl2p (Figure 6 and [54]). It is tempting to speculate that Sfl1p and Sfl2p may convey temperature regulation to the transcriptional network controlling biofilm formation.

*C. albicans* adaptation to temperature variation is one of the major critical traits of its ability to cause disease or to act as a commensal of warm-blooded species, as a temperature increase triggers hyphal development [2]. To date, three temperature-responsive transcription factors have been shown to play a role in *C. albicans* morphogenesis, Hsf1p [62], [63], Sfl2p [39], [40] and Hms1p [49]. Importantly, all three transcription factors are required for full virulence in different host/tissue models [39], [40], [49], [63], reinforcing the link between temperature adaptation and pathogenesis in *C. albicans*. The *HMS1* gene, encoding a basic helix-loop-helix (bHLH) transcription factor, has been recently isolated in a screen aimed at identifying transcription factors whose function is required for the *HSP90*- or high temperature-mediated filamentous growth [49]. Hms1p acts downstream of the Pho85p-Pcl1p cyclin-dependent kinase pathway but its function was still dependent upon cAMP-PKA signalling [49]. Interestingly, both Sfl1p and Sfl2p bind to the promoter of the *HMS1* gene, while Sfl2p downregulates its expression (Figure 6A), suggesting that activation of Sfl2p turns off the *HSP90*-dependent filamentation response (at least under the conditions used in the present study). Similar to Sfl2p, Hsf1p is an HSF-type transcription factor that induces transcription following a temperature increase, but, unlike *SFL1* and *SFL2*, *HSF1* is essential for viability [62]. Hsf1p is required for the expression of essential chaperones, including *HSP104*, *HSP90*, *HSP70* as well as other classical heat-shock protein (HSP)-encoding genes such as *HSP60*, *HSP78*, others [62]. Although carrying HSF-type domains in their primary protein sequences and sharing relatively high sequence similarity levels with Hsf1p, speculating a role in the transcriptional regulation of HSP (or HSP-related) genes, the Sfl1p and Sfl2p binding targets did not show any significant enrichment of functional categories pertaining to the heat-shock response pathway (e.g. protein folding/refolding), including HSPs and chaperones (Figure 2C). This may have important evolutionary implications as it might reflect specific needs of *C. albicans* to efficiently act as an opportunistic yeast of warm-blooded animals through converting temperature-sensing inputs into a morphogenesis programming output using HSF-type regulators like Sfl1p and Sfl2p. Nevertheless, we detected Sfl1p and Sfl2p binding at the promoter of the *HSP104*, *HSP70* and *SIS1* genes (binding intensity below algorithm threshold used for *HSP70*), suggesting that a reminiscent classical heat-shock response may have been retained in Sfl1p and Sfl2p. It is intriguing that one of the two potential binding motifs of Sfl1p (Figure 8A), 5′-TtCtaGaA-3′, is strikingly similar to the *S. cerevisiae* Hsf1p motif [64], [65], in line with the hypothesis that transcriptional rewiring affected the regulation of the heat shock response and temperature adaptation between *S. cerevisiae* and *C. albicans*.

It is worth noting that the predicted protein sequences of Sfl1p and Sfl2p are highly similar to those of *S. cerevisiae* Sfl1p and Mga1p. The *MGA1* gene has been initially isolated as a multicopy suppressor of both the *snf2*Δ (component of the SWI/SNF remodelling complex, also known as *gam1*) [66] and the *mep1*Δ/*mep1*Δ *mep2*Δ/*mep2*Δ (encoding ammonium permeases) filamentous defect [67] mutations in *S. cerevisiae*. Interestingly, Mga1p was shown to act as a master regulator of *S. cerevisiae* pseudohyphal development through direct transcriptional control of key genes involved in morphogenesis [68]. Many intriguing functional similarities exist between Sfl2p and *S. cerevisiae* Mga1p, although either *SFL1* or *SFL2* could complement an *sfl1*Δ mutation and *SFL2* could not complement the pseudohyphal growth defect of an *mga1*Δ mutant [39]. First, both proteins recognize similar DNA binding motifs (5′-AtAGAACA-3′ for Mga1p [33] and 5′-ANATAGAA-3′ for Sfl2p (Figure 8)). Second, both transcription factors bind to the promoter of orthologous genes (Sc*PHD1* and Sc*SOK2*/Ca*EFG1*, *HMS1*, Sc*GAT2*/Ca*BRG1*, *MSB2*, *ACH1*, Sc*ENA1*/Ca*ENA21*, *GCN4*, *CUP9*, *TPO4*, ScSCW4/CaMP65, others; binding to some genes is below peak-finding algorithm threshold). Third, the regulatory networks to which they belong are intriguingly similar: Mga1p establishes cross talks with major regulators of *S. cerevisiae* pseudohyphal growth including Phd1p, Sok2p (Efg1p orthologs), Flo8p and Tec1p, as in the case of Sfl2p (Figure 6) [39], [68]. Fourth, overexpression of *MGA1* and *SFL2* is sufficient to induce morphogenesis in the respective species under conditions that do not promote filamentation [39], [68]. Fifth, Sfl2p requires *EFG1* and *FLO8* to induce filamentation under specific conditions (Figure 7B and [39]) and we show here that Efg1p co-immunoprecipitates with Sfl2p (Figure 9B). Similarly, Mga1p requires a functional *FLO8* gene for its ability to bind DNA and Mga1p and Flo8p interact with each other [68]. We suggest that transcriptional rewiring may have affected the functions of Sfl2p and Mga1p in their respective species: In diploid *S. cerevisiae* cells, Mga1p responds to nitrogen limitation to turn on pseudohyphal growth, whereas in *C. albicans* Sfl2p responds to temperature increase to induce hyphal development.

## Materials and Methods

### Strains and growth media

The *C. albicans* strains used in this study are listed in Table 1. Depending on experimental conditions, *C. albicans* strains were grown in YPD (1% yeast extract, 2% peptone, and 1% dextrose), YP (1% yeast extract, 2% peptone) supplemented with 10% Fetal Bovine Serum (FBS), SD (synthetic dextrose, 0.67% yeast nitrogen base (YNB; Difco) with 2% glucose) [69] supplemented if necessary with arginine, histidine or uridine (20 mg/l each and 2% agar for growth on solid medium), SC (synthetic complete) or Lee's medium supplemented or not with methionine [70]. Expression from the tetracycline-inducible promoter (P*_TET_*) was achieved through addition of 3 µg/ml anhydrotetracycline (ATc - Fisher Bioblock Scientific) in YPD at 30°C [41]. ATc-containing cultures were maintained in the dark as ATc is light sensitive. *Escherichia coli* strains TOP10 (Invitrogen) or DH5α were used for DNA cloning and maintenance of the plasmid constructs.

### Plasmid construction and generation of epitope-tagged or mutant strains

All *C. albicans* transformation experiments used the lithium-acetate transformation protocol of Walther and Wendland [71] and selection of transformants for uridine or histidine prototrophy (when using the *URA3* or the *HIS1* markers, respectively) or Nourseothricine resistance (when using the *SAT1* marker) [72]. Plasmid pCaMPY-3xHA and the SGY243 strains expressing the *CAP1-HA_3_* allele or carrying the empty vector (pCaEXP) were kindly provided by Dr Martine Raymond (Université de Montréal, Canada). Strains AVL12 and HLCEEFG1 (expressing EFG1-HA under the control of the endogenous promoter) were the kind gifts of Dr Joachim Ernst (Heinrich-Heine-Universität, Dusseldorf, Germany). We first attempted to generate epitope (HA_3_, triple hemagglutinin)-tagged strains expressing Sfl1-HA_3_ or Sfl2-HA_3_ under the control of their endogenous promoter at their chromosomal location. *SFL1*- or *SFL2*-tagging cassettes were PCR-amplified from plasmid pCaMPY-3×HA [73] using primers SFL1-HA-FWD (forward, Table S9 in Text S1, the lowercase sequence corresponds to positions +2316 to +2415 of the *SFL1* ORF) and SFL1-HA-REV (reverse, Table S9 in Text S1, the lowercase sequence corresponds to positions +2419 to +2518 of the *SFL1* ORF) or primers SFL2-HA-FWD (forward, Table S9 in Text S1, the lowercase sequence corresponds to positions +2043 to +2142 of the *SFL2* ORF) and SFL2-HA-REV (reverse, Table S9 in Text S1, the lowercase sequence corresponds to positions +2146 to +2245 of the *SFL2* ORF), which anneal specifically to the in-frame pCaMPY-3×HA vector sequences PET-up and PET-down (respective uppercase sequences in Table S9 in Text S1), as described previously [73]. The resulting fragments (1,853 bp), containing the *C. albicans URA3* marker flanked by direct repeats of the HA_3_-encoding sequences and 100 bp of sequences homologous to the 3′ end of the *SFL1 or SFL2* genes, were used to respectively transform *ura3*-deficient *sfl1*Δ/*SFL1* and *sfl2*Δ/*SFL2* heterozygous mutants, yielding strains CEC3075 and CEC3076, respectively (Table 1). Expression of the Sfl1p-HA_3_ and Sfl2p-HA_3_ fusions in strains CEC3075 and CEC3076 was not detectable by Western blot analyses, suggesting that integration of the tagging cassette at the 3′ untranslated regions of *SFL1* and *SFL2* had a knockdown effect. Despite many attempts, excision of the *URA3* marker through intramolecular recombination between the HA_3_ sequences was not successful. We rather observed 100% loss of the entire tagging cassette at the *SFL1* and *SFL2* loci. We therefore used the pCaEXP system to drive expression of the tagged *SFL1* and *SFL2* alleles at the *RPS1* locus [42]. The *SFL1-HA_3_* or *SFL2-HA_3_* fusions were PCR amplified from CEC3075 or CEC3076 genomic DNA, respectively, using primers SFL1-HA-CaEXP-FWD (forward, Table S9 in Text S1, introduces a *Bgl*II site [underlined]) or SFL2-HA-CaEXP-FWD (forward, Table S9 in Text S1, introduces a *Bgl*II site [underlined]), respectively, and primer HA-CaEXP-REV (reverse, Table S9 in Text S1, introduces sequentially a *Bgl*II site [underlined] and a TAA stop codon [in red lowercase letters]). The resulting fragments (*SFL1-HA_3_*, ∼2,600 bp; *SFL2-HA_3_*, ∼2,330 bp) were digested with *Bgl*II and cloned into the compatible *Bam*HI site of plasmid pCaEXP, generating plasmids pCaEXP-*SFL1-HA_3_* and pCaEXP-*SFL2-HA_3_*. Plasmids pCaEXP (empty vector, control), pCaEXP-*SFL1-HA_3_* and pCaEXP-*SFL2-HA_3_* were digested with *Stu*I for integration at the *RSP1* locus [42] and the resulting fragments were used to transform strains CEC1910 and CEC1503 (Table 1), respectively, to generate strains *sfl1*-CaEXP, *sfl1*-CaEXP-*SFL1-HA_3_*, *sfl2*-CaEXP and *sfl2*-CaEXP-*SFL2-HA_3_* (Table 1).

Construction of *C. albicans* knock-out mutants (Table 1) used PCR-generated *ARG4*, *HIS1*, *URA3* and *SAT1* disruption cassettes flanked by 100 base pairs of target homology region (primer sequences are listed in Table S9 in Text S1) as described by Gola *et al.* [74] and Schaub *et al.* [75]. Independent transformants were produced and the gene replacements were verified by PCR on whole yeast cells as described previously [74], [75]. If necessary, transformants were converted to uracil prototrophy using *Stu*I-linearized CIp10 [76]. Mutant strains carrying the pCIp-P*_TET_*-*SFL2* [41] plasmid (Table 1) were first transformed with the pNIMX construct as described in Chauvel *et al.* [41].

Construction of chromosomally TAP-tagged *SFL1* and *SFL2* alleles (Table 1) used PCR-generated tagging cassettes from plasmid pFA-TAP-HIS, a derivative of the pFA-GFP-tagging plasmid series [74] (primers are listed in Table S9 in Text S1, oligos # 50-53) followed by targeted homologous recombination at the 3′ untranslated regions of *SFL1* and *SFL2* to generate strains expressing C-terminally tagged Sfl1p (strains SFL1-TAP and AVL12-SFL1-TAP, Table 1) and Sfl2p (strains SFL2-TAP and AVL12-SFL2-TAP, Table 1) proteins.

### Total protein preparation and Western blotting

Total protein extracts were prepared from 24 OD_600_ units of strains expressing (*sfl1*-CaEXP-SFL1-HA, *sfl2*-CaEXP-SFL2-HA) or not (empty vector; *sfl1*-CaEXP, *sfl2*-CaEXP) the *SFL1-HA_3_* or *SFL2-HA_3_* alleles (Table 1) grown overnight in SD medium (P*_MET3_*-inducing conditions). Cultured cells were centrifuged at 3,500 rpm during 5 min at room temperature and the pellets were resuspended in 150 µl of ice-cold TE buffer (10 mM Tris, [pH 7.5], 1.5 mM EDTA) supplemented with a protease inhibitor cocktail (Roche) and 1.5 mM phenylmethylsulfonyl fluoride (PMSF) then transferred to 1.5-ml tubes. The equivalent of 100 µl ice-cold glass beads was added to each tube and the suspensions were vortexed 5 times during 1 minute with 1-min incubations on ice in between. The extracts were clarified by centrifugation at 5,000 rpm during 1 min, boiled for 1 min and separated (25 µl) by electrophoresis on a sodium dodecyl sulfate-8% polyacrylamide gel. Proteins were electrophoretically transferred to nitrocellulose membranes. The membranes were incubated with a mouse anti-HA monoclonal antibody (12CA5; Roche) for 1 h at a dilution of 1∶1,000, followed by incubation with a horseradish peroxidase-conjugated secondary antibody (Sigma) during 30 min, washed, and developed with enhanced chemiluminescent detection reagents (ECL kit, GE Healthcare).

### Microscopy and image analyses

Cells were observed with a Leica DM RXA microscope (Leica Microsystems). Images were captured with a Hamamatsu ORCA II-ER cooled CCD camera, using the Openlab software version 3.5.1 (Improvision Inc.).

### ChIP-Seq, data preprocessing and analyses

Two independent cultures of strains *sfl1*-CaEXP or *sfl2*-CaEXP (untagged; control strains) and *sfl1*-CaEXP-*SFL1-HA_3_* or *sfl2*-CaEXP-*SFL2-HA_3_* (tagged strains) (Table 1) were grown overnight in 2 ml YPD at 30°C, diluted to an OD_600_ of 0.3 in Lee's medium deprived of methionine and cysteine (to induce P*_MET3_*) and grown during 4 hours at 37°C (hyphae-inducing conditions). The subsequent steps of DNA cross-linking, DNA shearing and chromatin immunoprecipitation (ChIP) were conducted as described in Liu *et al.* [73], with some modifications. Briefly, cultures were treated with 1% formaldehyde (cross-linking) and snap-frozen in liquid nitrogen. Total cell extracts were prepared by bead beating using a FastPrep-24 instrument (MP Biomedicals) with 6 runs during 1 minute each at 6.0 m/sec and 1 minute on ice in between (these settings led to efficient breakage of hyphal cells). Preparation of soluble chromatin fragments was performed by sonicating the extracts 6 times during 20 sec at power 8 (knob position) for an output signal amplitude of 15 (Microns, Peak to Peak) using a probe sonicator (MSE), yielding ∼200-bp DNA fragments on average. The extracts were then incubated at 4°C overnight with a mouse monoclonal anti-HA antibody (Santa Cruz Biotech) coupled to magnetic beads (pan-mouse immunoglobulin G Dynabeads; Dynal Biotech, Brown Deer, WI). The concentration of the purified immunoprecipitated DNA was ranging between 0.2 ng/µl and 1.5 ng/µl in 50 µl TE (10 mM Tris [pH 8.0], 1 mM EDTA). Library construction (10 ng of the immunoprecipitated DNA were used, adaptor-DNA fragments ranging from 150 to 350 bp) was performed using the TruSeq DNA sample preparation kit as recommended by the manufacturer (Illumina), followed by quality control analyses using a Bioanalyzer 2100 instrument (Agilent Technologies). DNA library samples were indexed and pools of the Sfl1p (4 samples, both tagged and control) or Sfl2p (4 samples, both tagged and control) ChIP samples were loaded onto two lanes of an Illumina HiSeq2000 sequencer flow cell for single-read (51 base pairs per read) high-throughput sequencing. The resulting 51-nucleotide sequence reads (FASTQ files) were imported into the Galaxy NGS data analysis software (https://main.g2.bx.psu.edu/) and the tools implemented in Galaxy were used for further processing via workflows [77], [78]. Quality control analyses of the FASTQ files were performed using FastQC (version 0.10.0, Babraham Institute) and adaptor-contaminated sequences were trimmed. The reads were then mapped to the *C. albicans* assembly 21 genome using the Bowtie algorithm [79] and the files of mapped reads (BAM files) for the ChIP sample (2 biological replicates from samples *sfl1*-CaEXP-*SFL1-HA_3_* or *sfl2*-CaEXP-*SFL2-HA_3_*) and from the control (2 biological replicates from samples *sfl1*-CaEXP or *sfl2*-CaEXP) were processed using the command line version 1.4Orc2 of the Model-Based Analysis for ChIP-Seq (MACS) peak-finding algorithm [46] for peak finding with the following parameters: bandwidth = 250; mfold = 10,30; shiftsize = 100; P-value cutoff for Sfl1p peaks = 1e-14 and P-value cutoff for Sfl2p peaks = 1e-100. Replicates 1 and 2 from the two independently performed ChIP-Seq experiments were processed separately. Overlapping peak intervals (intersection) from replicates 1 and 2 of Sfl1p or Sfl2p binding data were generated using the Galaxy tool Intercept version 1.0.0 (https://main.g2.bx.psu.edu/). The complete Sfl1p and Sfl2p binding and expression datasets are provided in Tables S1–S8 in Text S1. The command line version of the PeakAnnotator (v 1.4) sub-package from the PeakAnalyzer suite of algorithms [80] was used to annotate the Sfl1p and Sfl2p binding peaks in Tables S1, S2, S4 and S5 in Text S1. The association of peaks to target genes was also conducted by human eye (Tables S3 and S6 in Text S1), based on the location of ORFs relative to binding peaks. We provide wiggle tracks with tag counts for every 10 bp segment (See Materials and Methods section entitled “Data accession numbers” below). Visualization of the ChIP-Seq results was conducted using the Integrated Genomics Viewer software [44], [45].

### ChIP-PCR assays

Thirty cycles of PCR with 15 seconds at 95°C, 15 seconds at 50°C and 40 seconds at 70°C were performed on independently generated ChIP samples (Figures 3 and 9A) in a 50-µl reaction volume with 1 µl (5%) of immunoprecipitated material. Primers were designed to assay binding enrichment approximately around ChIP-Seq peak summits (primer sequences are provided in Table S9 in Text S1). The *URA3* and *YAK1* ORFs were used as negative controls.

### RNA isolation for microarray experiments

Strains *sfl1*-CaEXP or *sfl2*-CaEXP (control strains, for subsequent Cy3 labeling) and *sfl1*-CaEXP-*SFL1-HA_3_* or *sfl2*-CaEXP-*SFL2-HA_3_* (test strain, for subsequent Cy5-labeling) (Table 1) were grown overnight in 2 ml YPD at 30°C. The next day, an aliquot of the overnight culture was used to inoculate 50 ml of Lee's medium deprived of methionine and cysteine to a starting OD_600_ of 0.3. This culture was grown for 4 hours at 37°C, cells were washed with diethyl pyrocarbonate (DEPC)-treated water, collected by centrifugation and pellets were immediately frozen and stored at −80°C until RNA isolation. Three independently obtained sets of cell cultures were used. RNA was isolated from frozen cell pellets using the hot-phenol method [81]. Briefly, cells were resuspended in 375 µl TES buffer (10 mM Tris [pH 7.5], 10 mM EDTA, 0.5% SDS) at room temperature, after which 375 µl acid Phenol∶Chloroform (5∶1, Amresco, Solon, OH) were added. Samples were then incubated for 1 hour at 65°C with vigorous vortexing during 20 sec each 10 min and subjected to centrifugation for 20 min at 14,000 rpm. The supernatants were transferred to new tubes containing 750 µl acid Phenol∶Chloroform (5∶1), mixed, and subjected to centrifugation at 14,000 rpm for 10 min. The aqueous phase was transferred to new tubes containing 750 µl Chloroform∶Isoamyl alcohol (24∶1, Interchim, Montluçon, France), mixed and centrifuged at 14,000 rpm during 10 min. RNA was precipitated from the resulting aqueous layer by mixing that portion in new tubes with 1 ml 99% ethanol (pre-cooled at −20°C) and 37 µl of 3 M sodium acetate [pH 5.0] and subjecting the mixture to centrifugation at 14,000 rpm for 40 min at 4°C. The supernatants were removed, the pellet was resuspended in 500 µl 70% ethanol, and the RNA was collected by centrifugation at 14,000 rpm for 20 min at 4°C. The supernatants were again removed, and the RNA was resuspended in 150 to 300 µl DEPC-treated water. The RNA was stored at −80°C until needed.

### First-strand cDNA synthesis and microarray hybridization

Prior to first-strand cDNA synthesis, the purity and concentration of RNA samples were determined from A260/A280 readings (NanoVue Plus, GE Healthcare) and RNA integrity was determined by a Bioanalyzer 2100 instrument (Agilent Technologies) per the manufacturer's instructions (RNA concentration was ranging between 7.92 and 10.48 µg/µl). First-strand cDNA was synthesized from 20 µg total RNA, using the Superscript III indirect cDNA labeling system (Invitrogen) with the following minor modifications to the manufacturers' instructions. Briefly, the Qiagen PCR Purification kit was used to remove unincorporated aminoallyl-dUTP and free amines with substitution of the Qiagen-supplied buffers with phosphate wash (5 mM Phosphate buffer [K_2_HPO_4_/KH_2_PO_4_O_4_] [pH 8.0], 80% ethanol) and elution (4 mM Phosphate buffer [K_2_HPO_4_/KH_2_PO_4_O_4_] [pH 8.5]) buffers. The purified first-strand cDNAs were subsequently labelled with the monoreactive Cy dye *N*-hydroxysuccinimide esters Cy3 (control, cDNA from strains *sfl1*-CaEXP or *sfl2*-CaEXP) and Cy5 (cDNA from strains *sfl1*-CaEXP-*SFL1-HA_3_* or *sfl2*-CaEXP-*SFL2-HA_3_*) (GE Healthcare) and the uncoupled dye was removed using the standard Qiagen PCR purification kit protocol. The Cy3- and Cy5-labeled cDNA lyophilized pellets were resuspended in 10 µl of DNase-free water then 2.5 µl and 12.5 µl of 10X blocking agent and 2X hybridization buffer (Agilent Technologies), respectively, were added. The resulting samples were mixed, incubated at 95°C during 3 min and snap cooled on ice during 1 min then hybridized to a *Candida albicans* expression array (Agilent Technologies) designed such that two nonoverlapping probe sets are targeting each of 6,105 *C. albicans* ORFs for a total of 15,744 probes, thereby allowing two independent measurements of the mRNA level for a given gene (The EMBL-European Bioinformatics Institute ArrayExpress platform accession number: A-MEXP-2142, http://www.ebi.ac.uk/arrayexpress/arrays/A-MEXP-2142).

### Gene expression microarray data analysis

Images of Cy5 and Cy3 fluorescence intensities were generated by scanning the expression arrays using an Axon Autoloader 4200AL scanner (Molecular Devices, Downington, PA). Images were subsequently analyzed with the GenePix Pro 6.1.0.2 software (Molecular Devices, Downington, PA). GenePix Results (GPR) files were imported into the Arraypipe 2.0 [82] or the GeneSpring (Agilent Technologies) softwares. Following spot filtering and bad spot flagging, global signal intensities were normalized using Loess normalization and replicate slides (n = 3) were combined and the P-values calculated using a standard Student's *t*-test.

### Quantitative RT-PCR analyses

Total RNA was prepared from strains CEC2001 (*sfl1*Δ/*sfl1*Δ) and CEC1997 (*sfl1*Δ/*sfl1*Δ P*_PCK1_-SFL1-TAP*) or CEC1535 (*sfl2*Δ/*sfl2*Δ) and CEC1509 (*sfl2*Δ/*sfl2*Δ P*_PCK1_-SFL2-TAP*) (Table 1) during a kinetics experiment (0 h, 2 h and 4 h) in YNB plus 2% casaminoacids (P*_PCK1_*-inducing conditions). Cells from 100 mL cultures were mechanically disrupted with glass beads using a Fastprep (MP Biomedicals) and total RNA was extracted using RNAeasy (QIAGEN) according to the manufacturer's instructions. The quality and quantity of the isolated RNA were determined using an Agilent 2100 Bioanalyzer. Before cDNA synthesis, total RNA samples were DNase-treated using the Turbo DNA-free kit (Ambion). 2 µg of total RNA were used to perform cDNA synthesis using Superscript II Reverse Transcriptase according to the manufacturer's instructions (Invitrogen). Quantitative PCR was carried out on a Mastercycler ep realplex (Eppendorf) with a 2X SYBR Green master mix (SYBR Green Power, Applied Biosystems). The oligonucleotide primers used are listed in Table S9 in Text S1 (oligos # 18–27). The reaction mixture contained 2.5 µM of each primer and 5 µL of cDNA at 1∶10, 1∶100 or 1∶1000 dilutions. Each sample was processed in triplicate. Relative expression levels were calculated using the delta-delta Ct (ΔΔCt) method, with *C. albicans* translation elongation factor *CEF3* transcript as a calibrator. The relative expression was calculated as 2^(Ct target – Ct *CEF3* CEC1509 or CEC1997) – (Ct target– Ct *CEF3* CEC1535 or CEC2001)^.

### Co-immunoprecipitation experiments

Strains co-expressing Sfl1p-TAP and Efg1p-HA or Sfl2p-TAP and Efg1p-HA (AVL12-SFL1-TAP or AVL12-SFL2-TAP, respectively, Table 1) together with the control strains SFL1-TAP, SFL2-TAP and AVL12-pHIS (Table 1) were grown during 4 h in 50 ml SC medium at 30°C or Lee's medium at 37°C prior to crosslinking with formaldehyde. Cells were lysed with glass beads and total extracts were prepared in 700 µl lysis buffer (50 mM HEPES-KOH pH 7.5, 140 mM NaCl, 1 mM EDTA, 1% Triton X-100, 0.1% Na-deoxycholate) then sonicated as described for the ChIP-Seq experiment. Immunoprecipitation was performed with 500 µl of clarified sonicated extracts and 40 µl of IgG-coated magnetic beads (Dynabeads Pan mouse IgG, Invitrogen), previously prehybed overnight with PBS-0.1% BSA. The beads were washed once with 1 ml lysis buffer and three times with lysis buffer supplemented with 150 mM NaCl. Reverse crosslinking was achieved by incubating beads at 100°C during 25 min in reverse–crosslinking buffer (2% SDS, 0.5 M 2-mercaptoethanol, 250 mM Tris, pH 8.8). The immunoprecipitates were resolved by electrophoresis on an 8% SDS-polyacrylamide gel. Proteins were electrophoretically transferred to nitrocellulose membranes. Blots were revealed with rat monoclonal anti-HA peroxidase conjugate - High Affinity (clone 3F10, Roche) for detection of co-immunoprecipitated Efg1p-HA or with Peroxydase-Anti-Peroxydase Soluble complex (Sigma Aldrich) for detection of immunoprecipitated Sfl1p-TAP and Sfl2p-TAP at a 1∶2000 dilution.

### Bioinformatic analyses

Gene Ontology functional enrichment analyses were conducted using the CGD Gene Ontology (GO) Term Finder tool (http://www.candidagenome.org/cgi-bin/GO/goTermFinder). The orf19 list of the Sfl1p and Sfl2p common targets or the orf19 list of the Sfl2p-specific targets was used as input for functional grouping. To decide which of the two ORFs sharing the same bound promoter are included among the GO-term finder input list, we selected those ORFs showing differential expression in Sfl1p and Sfl2p transcriptomics data (expression level fold-change ≥1.5, P-value ≤0.05). This led to a list of 110 (Sfl1p and Sfl2p common targets) and 73 (Sfl2p specific targets) genes for GO term enrichment analyses (Table 2). If some GO terms contained overlapping gene lists, the GO term with the largest number of genes or with the best significance score was selected. The P-value cutoff for considering a functional grouping enrichment was *P*≤0.05. For motif discovery analyses, peak summit location files generated by the MACS algorithm [46] were imported into the Galaxy NGS analysis pipeline and DNA sequences encompassing ±250 bp around peak summits in Sfl1p or Sfl2p data sets were extracted using the Extract Genomic DNA tool version 2.2.2. The resulting sequences were used as input for motif discovery using the SCOPE (Suite for Computational Identification of Promoter Elements, version 2.1.0) program (http://genie.dartmouth.edu/scope/) [56] or the Regulatory Sequence Analysis Tools ([RSAT] http://rsat.ulb.ac.be/rsat/) peak-motifs algorithm [55]. The parameters used in RSAT peak-motifs algorithm were as follows: oligo-analysis and position-analysis were selected; oligo length was 6 and 7; the Markov order (m) of the background model for oligo-analysis was set to automatically adapt to sequence length; the number of motifs per algorithm was 10 and both strands of the DNA sequence inputs were searched for motif discovery. For building a control set of sequences (that is sequences randomly chosen from the genome), we used the RSA tool “random genome fragments”. The parameters used in SCOPE were as follows: species selected was *C. albicans* (genome sequence available at www.broad.mit.edu/annotation/genome/);“fixed” was selected for the upstream sequence control set and both strands of the DNA sequence inputs were searched for motif discovery.

### Data accession numbers

ChIP-Seq and microarray data can be found at the Gene Expression Omnibus (http://www.ncbi.nlm.nih.gov/projects/geo/) or ArrayExpress (http://www.ebi.ac.uk/arrayexpress/) databases under series numbers GSE42886 or E-MEXP-3779, respectively.

## Supporting Information

Figure S1 **Characterization of strains carrying chromosomally tagged alleles of** ***SFL1*** **and** ***SFL2*** **.** (**A**) Strains SFL1-TAP (CEC1922), SFL2-TAP (CEC1918) and EFG1-HA (HLCEEFG1), carrying chromosomally tagged *SFL1* (tandem affinity purification tag, TAP), *SFL2* (tandem affinity purification tag, TAP) and *EFG1* (haemagglutinin tag, HA) alleles were grown in SC medium at 30°C or Lee's medium at 37°C during 4 h together with the SC5314 strain as a control (CTRL) prior to microscopic examination (40× magnification). (**B**) Western blot (WB) analyses of strains SFL1-TAP, SFL2-TAP (upper panel) and EFG1-HA (lower panel) together with the SC5314 control strain (CTRL). Strains were grown in SC medium at 30°C (30°C) or in Lee's medium at 37°C (37°C) during 4 h and total protein extracts were prepared then subjected to SDS-PAGE. Western blotting was performed using an anti-TAP antibody (SFL1-TAP and SFL2-TAP, Peroxydase-Anti-Peroxydase Soluble complex, Roche) or an anti HA antibody (EFG1-HA, Monoclonal Anti-HA peroxidase conjugate - High Affinity (clone 3F10), Roche). Positions of the molecular mass standards are indicated on the left (kDa). Antibody cross-reacting signals were used as a loading control (Loading Control).
(TIF)

Text S1 **Includes Tables S1–S9 and full description of Tables S1–S9.**
(XLSX)
