# Supplementary figures and images for: A Comprehensive Functional Portrait of Two Heat Shock Factor-Type Transcriptional Regulators Involved in Candida albicans Morphogenesis and Virulence
Source: PLoS Pathog. 2013 Aug 15;9(8):e1003519. doi: 10.1371/journal.ppat.1003519 (PMC3744398; doi:10.1371/journal.ppat.1003519)

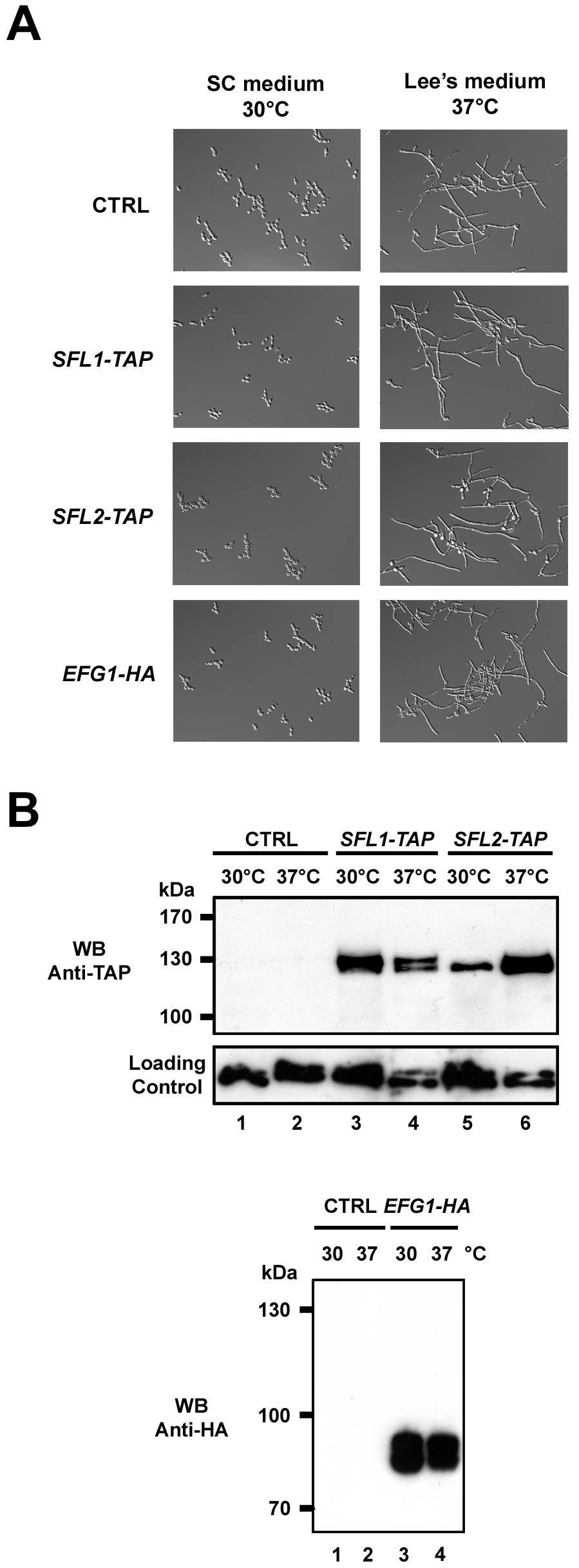

Supplement: Figure S1 — Characterization of strains carrying chromosomally tagged alleles of SFL1 and SFL2 . (A) Strains SFL1-TAP (CEC1922), SFL2-TAP (CEC1918) and EFG1-HA (HLCEEFG1), carrying chromosomally tagged SFL1 (tandem affinity purification tag, TAP), SFL2 (tandem affinity purification tag, TAP) and EFG1 (haemagglutinin tag, HA) alleles were grown in SC medium at 30°C or Lee's medium at 37°C during 4 h together with the SC5314 strain as a control (CTRL) prior to microscopic examination (40× magnification). (B) Western blot (WB) analyses of strains SFL1-TAP, SFL2-TAP (upper panel) and EFG1-HA (lower panel) together with the SC5314 control strain (CTRL). Strains were grown in SC medium at 30°C (30°C) or in Lee's medium at 37°C (37°C) during 4 h and total protein extracts were prepared then subjected to SDS-PAGE. Western blotting was performed using an anti-TAP antibody (SFL1-TAP and SFL2-TAP, Peroxydase-Anti-Peroxydase Soluble complex, Roche) or an anti HA antibody (EFG1-HA, Monoclonal Anti-HA peroxidase conjugate - High Affinity (clone 3F10), Roche). Positions of the molecular mass standards are indicated on the left (kDa). Antibody cross-reacting signals were used as a loading control (Loading Control). (TIF) [file ppat.1003519.s001.tif]
